# Multivariate Hydrogen-Bonded Organic Frameworks for Optimum Atmospheric Water Harvesting

**DOI:** 10.1021/acscentsci.5c01233

**Published:** 2025-09-04

**Authors:** Shan Liu, Lan Li, Xiang-Yu Gao, Rong Cao, Yue-Biao Zhang, Tian-Fu Liu

**Affiliations:** † State Key Laboratory of Structural Chemistry, Fujian Institute of Research on the Structure of Matter, Chinese Academy of Sciences, Fuzhou 350002, China; § School of Physical Science and Technology, Shanghai Key Laboratory of High-Resolution Electron Microscopy, ShanghaiTech University, State Key Laboratory of Advanced Medical Materials and Devices, 387433 ShanghaiTech University, Shanghai 201210, China; ‡ University of Chinese Academy of Sciences, Beijing 100049, China; # College of Materials and Chemistry, 92270 China Jiliang University, Hangzhou 310018, China

## Abstract

Hydrogen-bonded organic frameworks (HOFs) offer atomic-precision platforms for probing water adsorption, yet monotonic building units often fail to meet the multifaceted demands of atmospheric water harvesting (AWH). In this study, a multivariate (MTV) strategy is employed to tune adsorption onset, work capacity, and cycling stability in HOFs. Introducing amino groups in controlled ratios creates a balance between hydrophilic sites and dynamic confinement within ordered frameworks. Specifically, the parent HOF, PFC-76, was constructed from the organic linker [1,1′:4′,1″-terphenyl]-3,3″,5,5″-tetracarboxylic acid (TPTCA), which assembles into 2D honeycomb networks via carboxylic acid dimer synthons. Functionalizing TPTCA with amino groups modulates the framework’s packing and dynamic behavior. Single-crystal X-ray crystallography revealed sliding dynamics in PFC-76-NH_2_ during water adsorption, along with ordered water arrangements within the dynamic confinement spaces. Systematic variation of amino content (50%, 67%, and 80%) generated an atactic distribution of functional groups while maintaining crystallinity and porosity. This compositional tuning enhanced H_2_O uptake, optimized the adsorption inflection point, and delivered an outstanding cycling stability. The strategy demonstrates how precise control over functional group incorporation and framework dynamics can yield programmable performance in soft porous crystals for practical applications.

## Graphical abstract

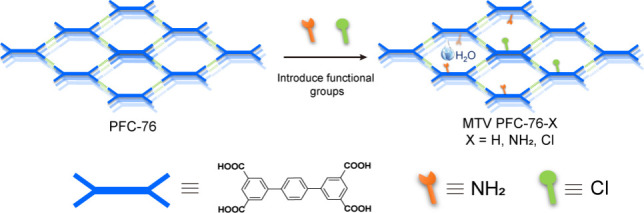

## Introduction

The scarcity of surface freshwater resources poses a significant challenge to the development of inland regions, despite the abundance of atmospheric water.^1,2^ Harvesting water from air has emerged as a promising technological solution.^3–9^ However, conventional dew collection technologies typically require a very high relative humidity, a condition difficult to achieve in arid environments. An ideal material for atmospheric water harvesting should exhibit the following characteristics: (i) a moderate and tunable adsorption inflection point during the adsorption phase (if the inflection point is too high, water uptake becomes inefficient under practical conditions, whereas if too low, excessive energy is needed for desorption; an optimal inflection point is around *P*/*P*_0_ = 0.45); (ii) high water uptake capacity to ensure efficient adsorption; (iii) minimal or no hysteresis during desorption, allowing for the easy and rapid release of captured water with low energy input; (iv) long-term cycling stability.^10–16^

Traditional hygroscopic materials, such as silica gel and zeolites, often face limitations in adsorption capacity and require a high desorption energy. Recently, metal–organic frameworks (MOFs) have shown great promise for water adsorption. Notable examples include the MOF-303/MOF-333 series, which offer tunable adsorption inflection points by adjusting the ratios of hydrophilic ligands, such as H_2_PZDC and H_2_FDC.^13,14^ These MOFs demonstrate high water uptake and excellent cycling stability. However, achieving broad tunability of these inflection points remains a significant challenge. Furthermore, competitive coordination between water molecules and metal nodes can compromise the structural stability of MOFs, particularly under cyclic adsorption–desorption conditions. Consequently, the development of metal-free water harvesting materials has attracted an increasing level of interest.

Hydrogen-bonded organic frameworks (HOFs) are a class of porous materials formed by the self-assembly of organic building units through noncovalent interactions, such as hydrogen bonding and π–π stacking interactions.^17–26^ They offer several advantages, including structural flexibility, low density, solution processability, and ease of recycling. Despite these benefits, systematic studies on the use of HOFs for water adsorption are largely untapped.^27,28^ In particular, no established strategies exist for tuning the inflection point of water adsorption isotherms, optimizing adsorption capacity, and achieving a balanced trade-off between these two key parameters in the HOF field. Inspired by multivariate MOFs (MTV-MOFs)^29–32^ and covalent organic frameworks (MTV-COFs),^33^ which incorporate various functional groups into their structures to modulate adsorption behavior, we aim to develop multivariate HOFs (MTV-HOFs)^21,34,35^ and investigate their water adsorption properties. However, this approach presents several challenges for molecular assemblies based on hydrogen bonds, including the following: (i) introducing new functional groups may alter the framework’s connectivity, resulting in a completely new structure; (ii) differences in monomer binding energies can sometimes drive the self-assembly of individual components into separate frameworks, rather than integrating multiple building blocks into a single structure; (iii) the spatial distribution of monomers within MTV-HOF crystals may be nonuniform and difficult to characterize.

To address the challenges mentioned above, it is crucial to introduce additional interactions between components to maintain the structural isomorphism. Among these, π-π stacking interactions are widely recognized as key stabilizing factors for HOF architectures. In this study, we selected the organic linker [1,1′:4′,1″-terphenyl]-3,3″,5,5″-tetracarboxylic acid (TPTCA, Figure 1a) as the building block for constructing a prototype HOF, owing to its planar, conjugated backbone and multitopic carboxylic groups. These features not only enable strong π-π interactions but also provide high connectivity (up to eight hydrogen bonds), ensuring a robust framework. To modulate the water adsorption properties, linkers containing -NH_2_ and -Cl functional groups were introduced by substituting a hydrogen atom on the central phenyl ring of TPTCA, resulting in TPTCA-NH_2_ and TPTCA-Cl. The flexibility of the dihedral angles α and β of TPTCA (Figure 1a) determines the stable conformation when incorporated into HOFs. The energy landscape^36–39^ of TPTCA (Figures 1b–1d) reveals that TPTCA-X (-X = -H, -NH_2_, -Cl) adopts multiple stable conformations, which facilitates the stabilization of molecules bearing diverse functional groups within an ordered structure. Due to variations in monomer H-bonding ability of the individual components, the monomer distribution within the HOF deviates significantly from the uniformity expected in an ideal solid solution.^34^ Nevertheless, incorporating large π-conjugated molecules into the framework has proven to be an effective strategy to overcome this challenge, ultimately enabling the successful construction of robust MTV-HOFs.^21,34,35^

**Figure 1:**
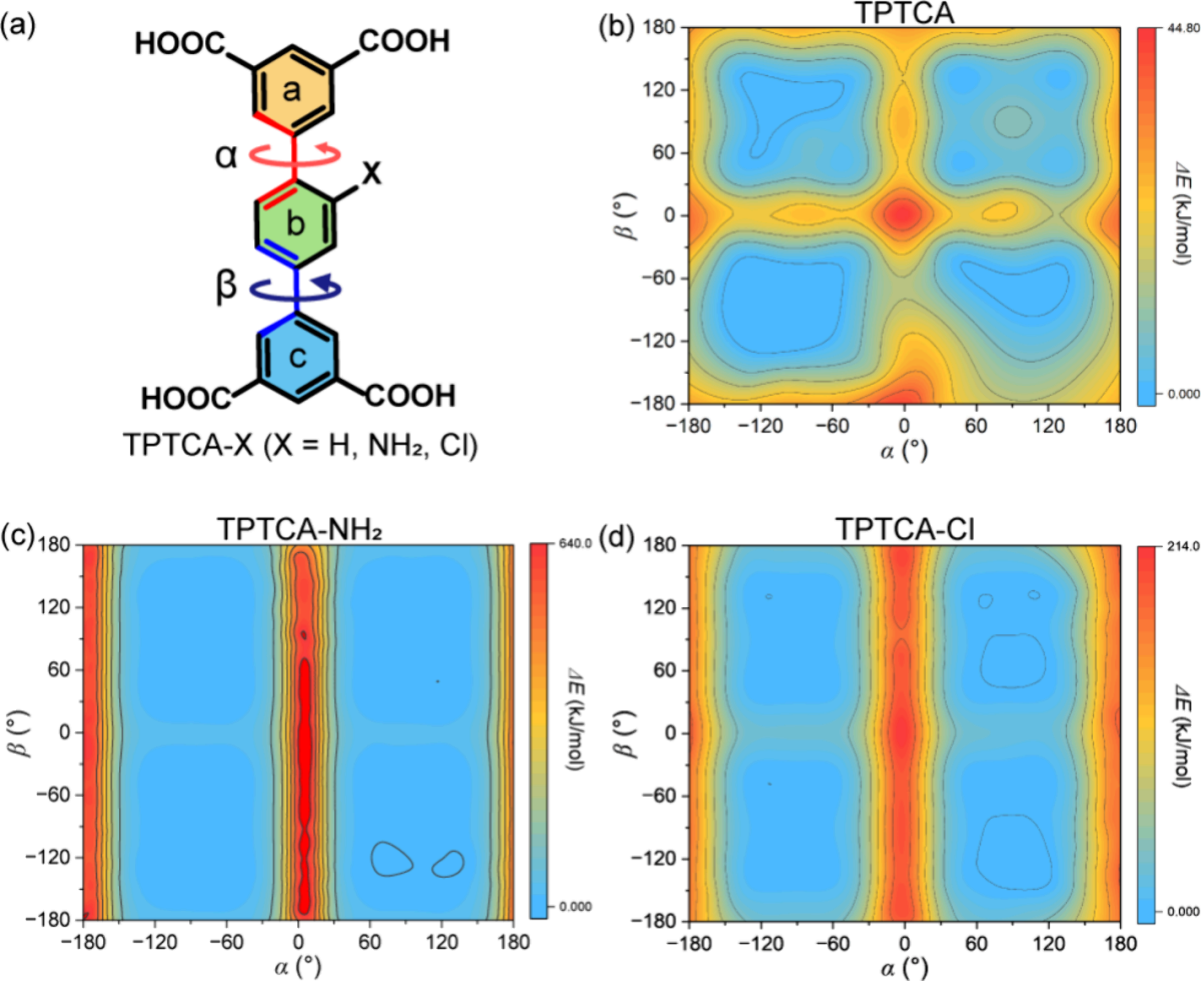
(a) The dihedral angles between phenyl rings (α for plane a and b, β for plane b and c); and the rotational energy landscape of (b) TPTCA, (c) TPTCA-NH_2_, and (d) TPTCA-Cl.

In this study, isostructural PFC-76-X (where X = H, NH_2_, and Cl; PFC = porous frameworks from FJIRSM, CAS) and a series of MTV-HOFs (PFC-76-NH_2_-*x* (*x* = 50%, 67%, and 80%), PFC-76-Cl-*y* (*y* = 50% and 67%), and PFC-76-NH_2_/Cl-67%) were successfully synthesized. Water adsorption experiments revealed that the isotherm shape, working capacity, and inflection points^16,40^ significantly varied with functional groups. Among these, PFC-76-NH_2_ exhibited the least hysteresis in the isotherm and an inflection point at a lower relative pressure. Single-crystal X-ray diffraction (SCXRD) and density functional theory (DFT) studies of the dehydrated and rehydrated phases (PFC-76-NH_2_ and -H_2_O) demonstrated that this behavior arises from the sliding of the layered network and the well-exposed adsorption sites, induced by the rotational motion of the linker during the water adsorption process. The MTV strategy enabled us to further enhance the working capacity of the materials. As a result, PFC-76-NH_2_-67% exhibited the highest water working capacity at 0.47 *P*/*P*_0_. This demonstrates that the MTV modular design strategy can be applied to HOF materials to achieve tailored properties, providing a new platform for water adsorption applications.

## Results and Discussion

### Crystal Structures of Functionalized HOFs

The needle-like crystalline PFC-76 was synthesized through self-assembling TPTCA linker in a mixed solvent of *N*,*N*-dimethylformamide (DMF) and acetic acid (AcOH) at 130 °C (Section S1 in the Supporting Information, SI).^41^ SCXRD structure analysis reveals that, in the as synthesized structure in PFC-76 (monoclinic *C*2/*c*, *a* = 16.24 Å, *b* = 26.57 Å, *c* = 7.21 Å, β = 114.56°, *V* = 2832.1 Å^3^, Table S1 in SI), each TPTCA building block interacts with four neighboring units via complementary O···O hydrogen bonds (2.61 Å) between carboxyl dimers, indicating a strong hydrogen bonding interaction (Figure 2a). PFC-76 has a one-dimensional hexagonal channel with dimensions of 10.3 × 11.4 Å^2^, and the angle θ of the hexagon is 121.54°. Fortunately, by introducing -NH_2_ and -Cl functional groups to the central phenyl of the TPTCA linker, high-quality crystals were obtained in the as-synthesized structure (PFC-76-NH_2_-as, triclinic *P*1̅, *a* = 7.87 Å, *b* = 13.68 Å, *c* = 14.21 Å, α = 111.60°, β = 94.78°, γ = 97.38°, *V* = 1395.86 Å^3^; PFC-76-Cl, monoclinic *C*2/*m*, *a* = 14.74 Å, *b* = 26.76 Å, *c* = 3.68 Å, β = 90.77°, *V* = 1451.7 Å^3^, see Figures S30–S31, Table S1 in SI). As shown in Figure 2a, the hydrogen-bond lengths, hexagonal channel sizes, and angle θ slightly changed in these structures (Figures 2b-2c). The layer of PFC-76 displays the AB packing mode with a distance of 3.54 Å (Figure 2). PFC-76-NH_2_-as adopts the same packing mode (AB, layer distance: 3.60 Å) as PFC-76, while the introduction of Cl groups changes the structure to an AA stacking mode (layer distance: 3.52 Å). δ_g_^inter^ (isosurfaces of 0.005 au) region calculated by independent gradient model based on Hirshfeld partition (IGMH) analysis^42–44^ revealed that the adjacent layers of PFC-76 and PFC-76-Cl are primarily stabilized by π-π interactions between the phenyls of TPTCA and TPTCA-Cl respectively (Figures S54–S55 in SI). Notably, for PFC-76-NH_2_-as, in addition to the π-π interactions, intermolecular H_NH_2__···O hydrogen bonding also contributes to the structure stability (Figure S56 in SI).

**Figure 2:**
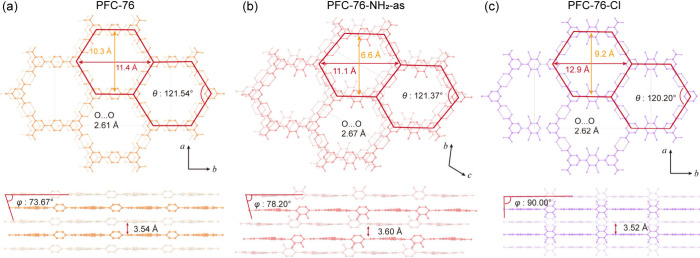
Stacking modes as well as interlayer distances and ϕ angles for (a) PFC-76, (b) PFC-76-NH_2_-as, and (c) PFC-76-Cl.

To quantify the change of the interlayer in PFC-76-X, ϕ angles of interlayers in topology were analyzed (Figure 2). For the AB stacking of PFC-76 and PFC-76-NH_2_-as, the ϕ angles are 73.67° and 78.20°, respectively. For the AA stacking of PFC-76-Cl, the ϕ angle is 90.00°. These variations can be attributed to subtle differences in steric hindrance and electrostatic repulsion, as revealed by the distinct dihedral angles α, β, and γ in these three structures (γ is the dihedral angle between a and c plane, Figure 1a, Figure S49, Table S3 in SI). The α, β, and γ angles of TPTCA (31.59°, 36.02°and 4.83°, respectively) are unstable conformation according to energy landscape (Figure 1b, Figure S51 in SI), which, however, were stabilized in PFC-76 by π-π interaction and hydrogen bonds. For PFC-76-NH_2_-as and -Cl, the organic linker with α, β and γ angles approximate the ideal conformation geometry on the energy landscape (Figures 1c and 1d, Figures S52–S53 in SI). Notably, the incorporation of -Cl groups aligns the a and c phenyl rings on the same plane in PFC-76-Cl with γ ≈ 0.

The powder X-ray diffraction (PXRD) patterns of PFC-76-X exhibit sharp reflection peaks with a full width at half-maximum (fwhm) < 0.27°, indicating the high crystallinity (Figure 3a). Additionally, PFC-76-X demonstrates excellent chemical stability, as evidenced by the retention of sharp PXRD reflection peaks after being soaked in pH 1 and 3 solvents (Figures S1–S2 in the SI). CO_2_ adsorption isotherms at 195 K reveal that PFC-76-NH_2_-as has the highest pore volume (*V*_p_) of 0.33 cm^3^ g^–1^ compared to those of PFC-76 and PFC-76-Cl (Figure 3b). This enhancement is attributed to the electrostatic interactions between the -NH_2_ groups and the dipole moments of CO_2_.

**Figure 3:**
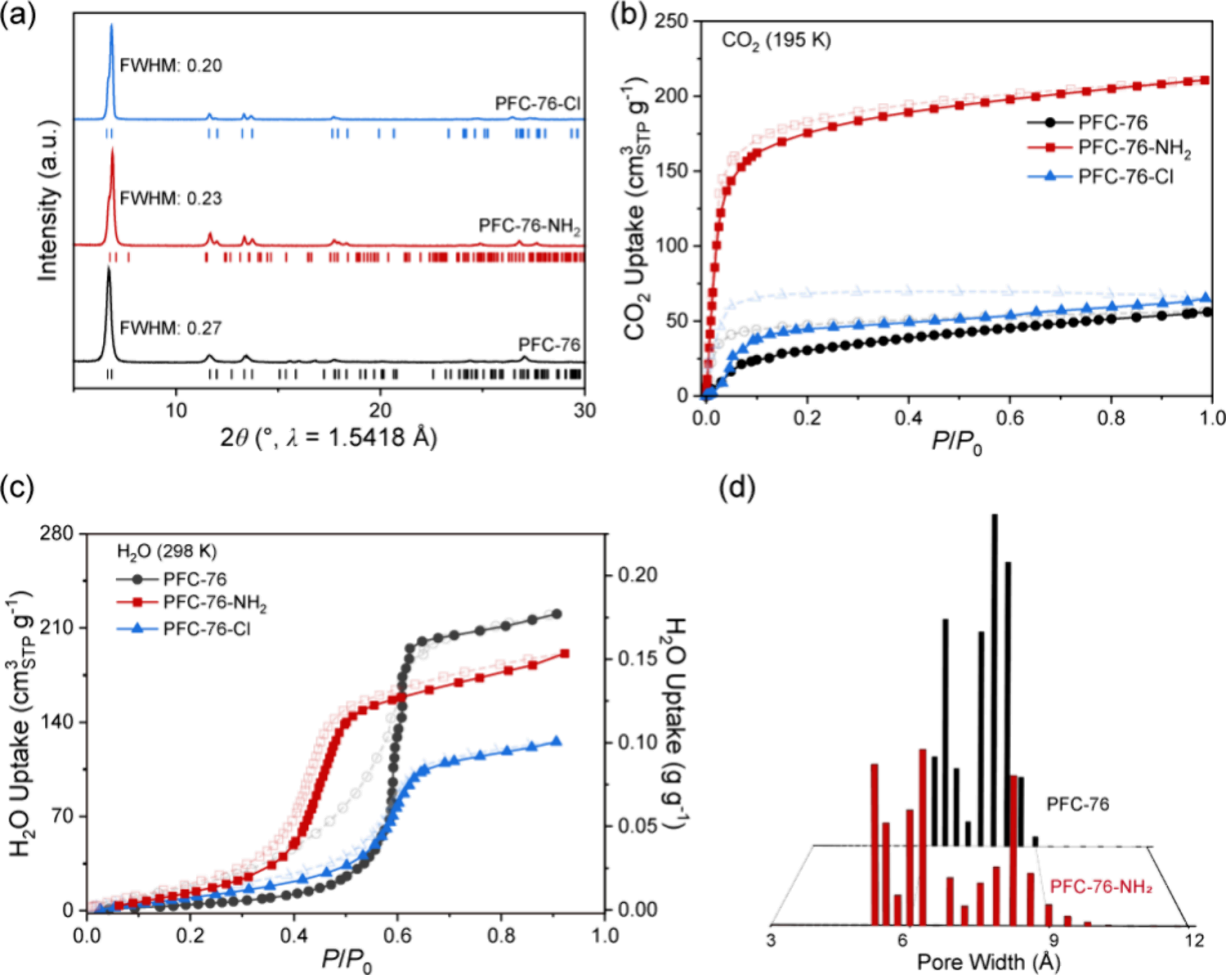
(a) PXRD patterns, (b) 195 K CO_2_ adsorption isotherms, (c) 298 K H_2_O adsorption isotherms, and (d) pore width distributions of PFC-76-X (X = H, NH_2_, Cl).

H_2_O adsorption isotherm of PFC-76 at 298 K exhibits a sigmoidal shape with a noticeable hysteresis loop at *P*/*P*_0_ ∼ 0.5 (Figure 3c). Notably, the inflection point of PFC-76 is *P*/*P*_0_ ∼ 0.6, reaching a working capacity up to 0.013 g g^–1^ (Table S2, Figure S40 in SI). The hysteresis phenomenon suggests that the water desorption process requires overcoming a substantial energy barrier to dissociate the hydrogen-bonded network of the guest water,^45^ which is unfavorable for efficient water release. Furthermore, cycling experiments conducted on regenerated PFC-76 revealed that only half of the overall uptake was retained, indicating poor recyclability (Figure S36 in SI).

Interestingly, the introduction of -NH_2_ and -Cl functional groups into PFC-76 resulted in materials exhibiting reversible water adsorption isotherms without hysteresis behavior. This phenomenon can be attributed to the reduction in pore width, which weakens the capillary condensation effect during water desorption. The pore width distribution, calculated from 273 K CO_2_ adsorption isotherm, shows that pores width with sizes <6 Å appeared after -NH_2_ and -Cl functionalization (Figure 3d, Figures S23 and S27 in SI).

Notably, PFC-76-NH_2_ exhibits a sharp water adsorption inflection point shifting to a low pressure of *P*/*P*_0_ ∼ 0.45, which is highly beneficial for water harvesting. This performance aligns well with the criteria for practical absorbents, which require efficiently capturing water molecules at low relative humidity and low energy barrier for the subsequent release.

### Atom Resolution Structure Revealed Water Adsorption Sites for PFC-76-NH_2_

To further reveal the adsorption sites in the structure, SCXRD data were collected on relative environment humidity PFC-76-NH_2_ (denoted as PFC-76-NH_2_, the structure was collected on atmosphere after activated) and rehydrated PFC-76-NH_2_ (denoted as PFC-76-NH_2_-H_2_O). Compared to PFC-76-NH_2_-as, PFC-76-NH_2_ displays the same *P*1̅ space group (*a* = 9.18 Å, *b* = 9.52 Å, *c* = 14.07 Å, α = 99.10°, β = 98.95°, γ = 117.04°, *V* = 1044.6 Å^3^, Figure S32, Table S1 in SI). In PFC-76-NH_2_-H_2_O, water molecules induce a more ordered structure with the *I*2/*a* space group (*a* = 7.52 Å, *b* = 26.63 Å, *c* = 14.92 Å, α = γ = 90.00°, β = 90.14°, *V* = 1044.6 Å^3^, Figure S33, Table S1 in SI). Dynamic transformation behaviors were observed, in which the initial PFC-76-NH_2_-as structure underwent pore shrinkage upon activation (PFC-76-NH_2_) and subsequently showed pore expansion upon water adsorption (PFC-76-NH_2_-H_2_O).

PFC-76-NH_2_ also exhibits hexagonal channel with sizes of approximately 8.7 × 13.9 Å^2^, AB interlayer stacking mode, slightly increased interlayer distance, and decreased angle ϕ compared to as-synthesized PFC-76-NH_2_-as (Figure 4a). As shown in Figure 4b, the size of the PFC-76-NH_2_-H_2_O hexagonal channel is 10.0 × 11.4 Å^2^, and the layer stacking maintains an AB packing mode with shortened layer distance (3.61 Å) and expanded angle ϕ (62.17°). The dihedral angles of building block in both the activated phases (α = 43.51°, β = −15.81°, and γ = 30.51°, Table S3 in SI) and the hydrated phase (α = 40.30°, β = −42.31°, and γ = 1.17°, Table S3 in SI) adopt energetically stable conformations according to the energy landscape (Figure 1c, Figure S52 in SI). The structure of PFC-76-NH_2_ is significantly altered compared to that of pristine PFC-76-NH_2_-as. Structure fragment analysis (Figures S34 and S35 in SI) reveals that the rotation of TPTCA-NH_2_, as reflected by the dihedral angles α, β, and γ (Table S3 in SI), critically influences the conformation, leading to substantial changes in the framework and pore architecture.

**Figure 4:**
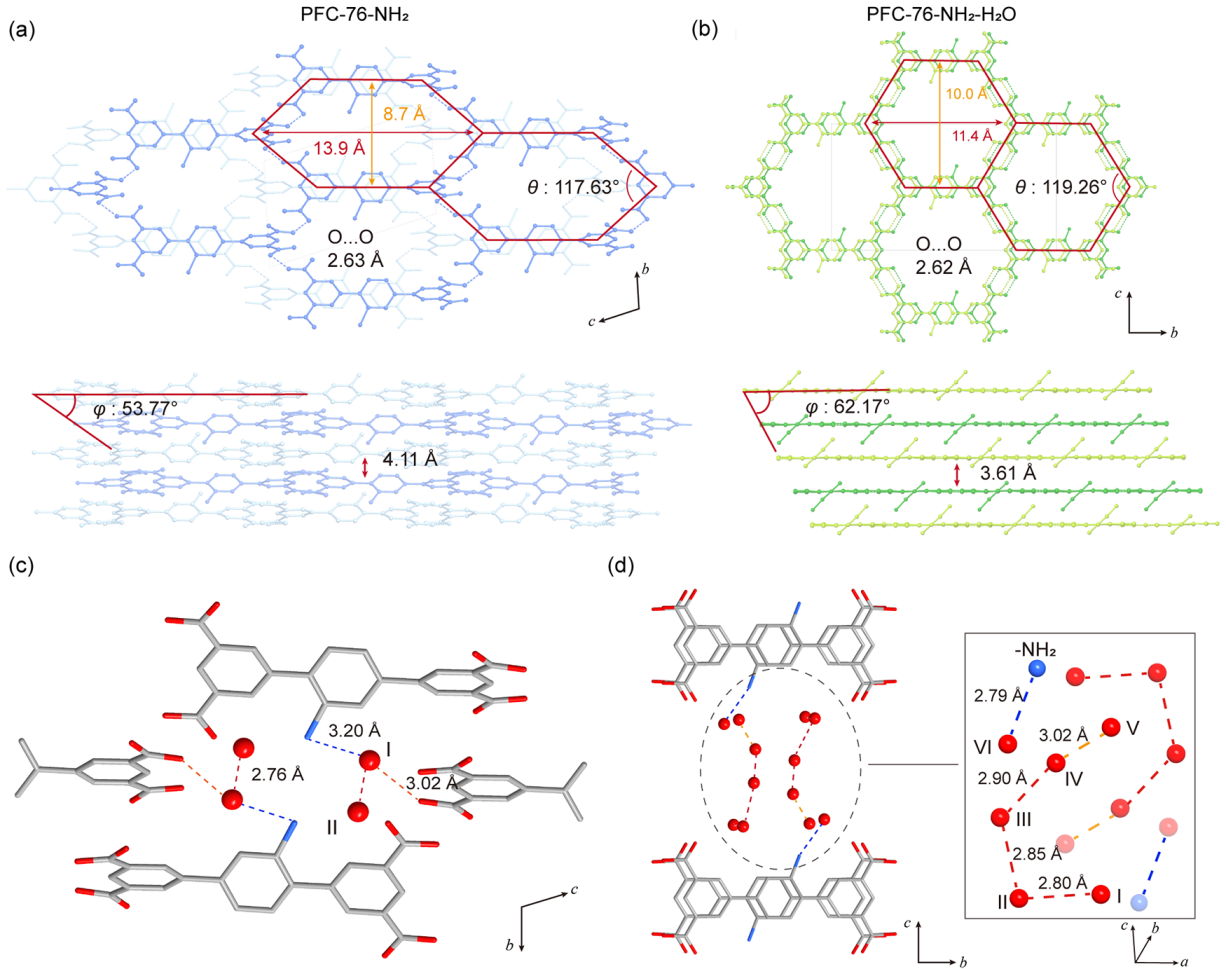
Structural topology of (a) PFC-76-NH_2_ and (b) PFC-76-NH_2_-H_2_O. The water clusters and the interaction between guest H_2_O molecules and -NH_2_ groups of the host framework in (c) PFC-76-NH_2_ and (d) -H_2_O.

SCXRD analysis provides precise structural information about the host framework of the PFC-76-NH_2_ and PFC-76-NH_2_-H_2_O phases, and the oxygen atoms of water molecules were clearly identified. However, owing to the disorder distribution of the -NH_2_ functional groups, the precise determination of water adsorption sites was challenging. Therefore, the H_2_O adsorption sites were further established using density functional theory (DFT) calculations (, SI), based on an idealized model structure with only one -NH_2_ group in the PFC-76-NH_2_ and PFC-76-NH_2_-H_2_O structure. DFT optimization of the PFC-76-NH_2_ phase identifies two primary water adsorption sites, denoted as sites I and II, each forming a strong hydrogen bond with an O···O distance of 2.76 Å (Figure 4c). Water at site I forms weak hydrogen bonds with the TPTCA-NH_2_ group with the O_I_···O_COOH_- and O_I_···N_NH2_ distances of 3.02 and 3.20 Å respectively. These interactions, involving both neighboring water molecules and the host framework functional groups, facilitate the water uptake and shift the adsorption inflection point toward lower relative humidity. As the water uptake increases to 6 mol/mol (Figure S37 in SI), the framework undergoes a layer-sliding to accommodate more water molecules, forming the hydrated phase PFC-76-NH_2_-H_2_O with more water sites denoted as I–VI. DFT-optimized structures reveal that water molecules at sites I–V form hydrogen-bonded networks with O···O distances of 2.80–3.02 Å^46^ (Figure 4d), while water at site VI forms a strong hydrogen bond with an -NH_2_ group on the host backbone, featuring an N···O_VI_ distance of 2.79 Å (Figure 4d). The intermolecular region (δ_g_^inter^: isosurfaces of 0.005 au) conducted using the IGMH method revealed that the hydrogen bond interactions exist between both host–guest and guest–guest, which are consistent with the DFT optimized structure analysis (Figures S57–S61 in SI). Compared with the structure of PFC-76-NH_2_, the interaction between guest molecules and host–guest rearranged and become stronger upon H_2_O adsorption.

### Preparation and Characterization of MTV-HOFs

Although PFC-76-NH_2_ effectively addresses the challenge of appropriate adsorption inflection points and desorption hysteresis, its overall H_2_O uptake was diminished compared with PFC-76, which inspired us to achieve a balanced trade-off between these parameters through regulation of the ratios of functional groups through the MTV strategy. Herein, by tuning the feeding ratio of TPTCA and TPTCA-NH_2_ in the reaction mixture, a series of binary MTV-HOFs with varied ratios named PFC-76-NH_2_-*x* (where *x* = 50%, 67%, 80%, based on the feeding mass ratio of TPTCA- NH_2_ in reaction), were successfully synthesized ( in SI). PXRD patterns of MTV-PFC-76-NH_2_ indicate that the obtained MTV-HOFs series are highly crystalline materials with the same topology as PFC-76-NH_2_ (Figure 5a). In the following discussion, PFC-76-NH_2_-67% is selected as a representative case for a detailed investigation. ^1^H NMR measurements spectra, as shown in Figure 5b, indicated the coexistence of the TPTCA linkers and TPTCA-NH_2_ in the PFC-76-NH_2_-67% materials, and the linker ratios incorporated in HOFs nearly approached the feeding mass ratios. The amino groups in PFC-76-NH_2_-67% were found to be partially protonated, and reversible deprotonation could be achieved in pH 9 aqueous solution (details in Figures S10, S11, S13 and S14 in SI). Additionally, energy dispersive spectrometry (EDS) mapping in the PFC-76-NH_2_-67% crystal revealed that C and N elements are uniformly distributed throughout the entire particle (Figure S7 in SI), suggesting that the TPTCA-NH_2_ and TPTCA linkers are evenly arranged within a single framework. The chemical stability of PFC-76-NH_2_-67% is proved by the sharp peak in the PXRD pattern in aqueous solutions at pH 3 and pH 1 (Figures S1–S2 in SI). The porosity of the MTV samples was evaluated via 298 K CO_2_ adsorption, which revealed a gradual increase in the CO_2_ uptake capacity with higher -NH_2_ ratios, attributable to the strong binding affinity between -NH_2_ groups and the CO_2_ guests (Figures S18, S24 and S28 in SI). By contrast, the 298 K CO_2_ uptake of the physical mixture containing 67% PFC-76-NH_2_ and 33% PFC-76 is lower than that of PFC-76-NH_2_-67% (Figure S22 in SI). The pore width distribution calculated from the 273 K CO_2_ adsorption isotherm shows that all samples in the MTV series have micropores smaller than 6 Å (Figure S25 in SI). *In-situ* diffuse reflectance infrared Fourier transform (FT-IR) spectroscopy revealed that PFC-76-NH_2_-67% forms hydrogen bonds during water adsorption (Figure S6 in the SI). Introduction of -NH_2_ groups significantly increases the hydrophilicity of the structure with the contact angle (Figure S9 in SI) decreasing from 76° (for PFC-76) to 30.5° (for PFC-76-NH_2_-67%). Further increasing the feeding ratio of -NH_2_, the contact angle of PFC-76-NH_2_ became undetectable due to the complete wetting of the surface by water droplets.

**Figure 5:**
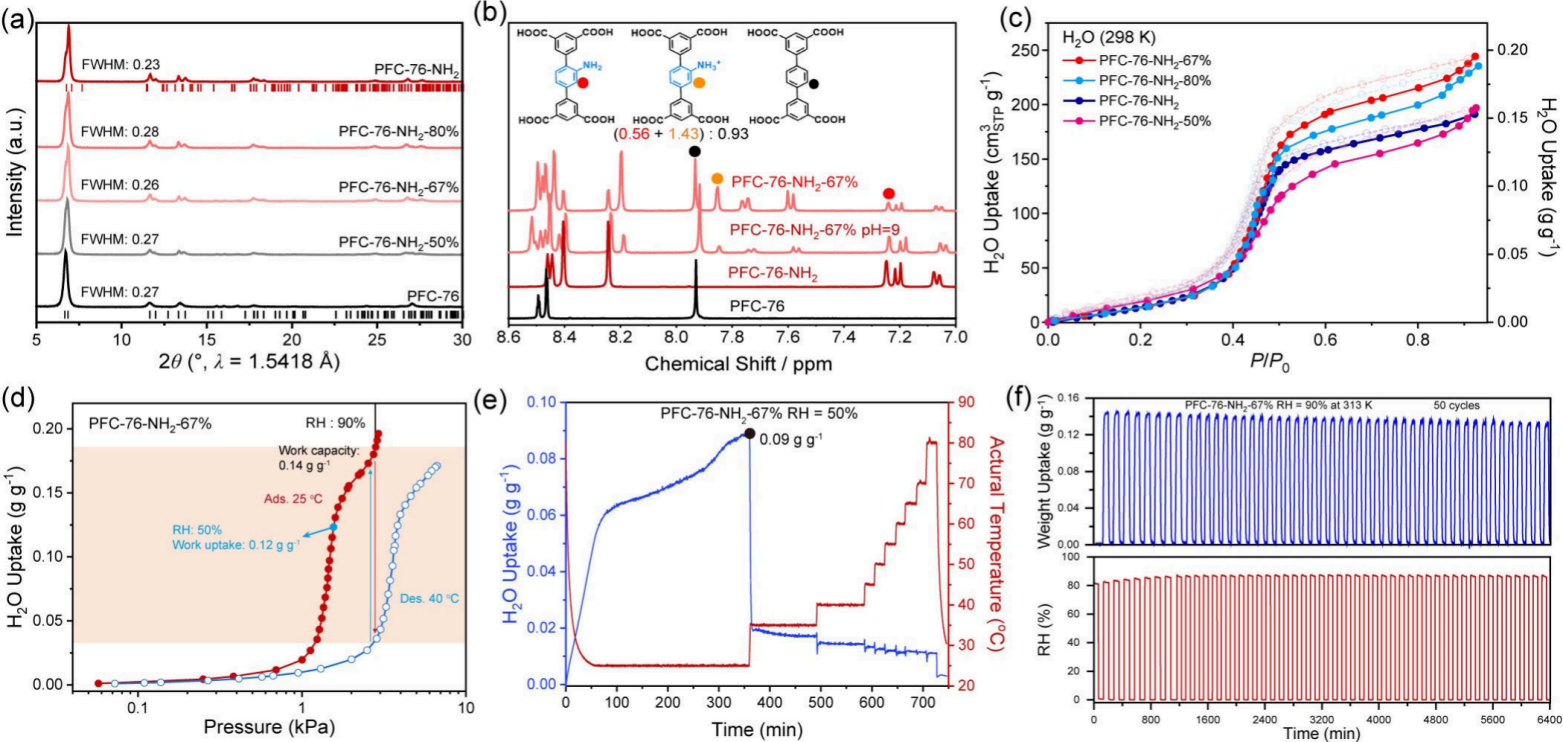
(a) PXRD patterns of the PFC-76-NH_2_-*x* series. (b) ^1^H NMR of PFC-76-NH_2_-67%. (c) Water adsorption of the PFC-76-NH_2_-x series at 298 K. (d) Working capacity of PFC-76-NH_2_-67% during H_2_O adsorption at 298 K and desorption at 313 K under 90% RH. (e) DVS test of PFC-76-NH_2_-67% at 50% RH after water adsorption saturation at different temperatures. (f) 50 cycles of the water uptake profile versus relative humidity for PFC-76-NH_2_-67% at 40 °C.

### MTV Strategy Enhanced Water Capture and High Stability

Static H_2_O adsorption analysis at 298 K for the MTV series (Figure 5c) revealed that the -NH_2_ functional groups induces an inflection point in the H_2_O adsorption shifting to lower pressure (*P*/*P*_0_ < 0.48), attributed to the interaction between -NH_2_ and H_2_O guests. PFC-76-NH_2_-67% exhibited the highest water uptake capacity (0.13 g g^–1^, Table S2, and Figure S40 in SI). The heat of adsorption for MTV PFC-76-NH_2_-*x* ranges from 40 to 60 kJ/mol, suggesting the presence of both host–guest and guest–guest interactions between the framework and H_2_O, with suitable energy consumption (Figures S41–S48 in SI).^47–49^

Pore size and functional group play crucial roles in water adsorption. Although PFC-76-NH_2_ offers the highest density of water adsorption sites, abundant bulky -NH_2_ groups reduce the pore volume and thereby diminish the working capacity. In contrast, PFC-76-NH_2_-67% with an optimized balance of pore size and adsorption sites achieves the highest water uptake within a narrow humidity range without obvious desorption hysteresis, thus fulfilling the criteria as an effective water absorbent. This MTV strategy provides valuable insights into the design principles of efficient absorbents for water harvest.

To further reveal the above structure–property relationship, MTV PFC-76-Cl-*y* (*y* = 50%, 67%, based on the feeding mass ratio of TPTCA-Cl in synthesized, Section S1 in SI) and PFC-76-NH_2_/Cl-67% (the combination of 67% TPTCA-NH_2_ and 33% TPTCA-Cl in framework) were synthesized (Section S1 in SI). Sharp PXRD patterns (Figure S4 in SI) indicate the high crystallinity of these materials, and EDS mapping of Cl in a PFC-76-Cl-67% crystal further confirms the successful incorporation (Figure S8 in SI). ^1^H NMR indicated the coexistence of TPTCA and TPTVA-Cl linkers in the PFC-76-Cl-67% material, and the linker ratios incorporated in HOFs nearly approached the feeding mass ratios (Figures S10, S12, S15–S17 in SI). 298, 273, and 195 K CO_2_ adsorption isotherms confirm the porous characteristics of MTV PFC-76-Cl-*y* and PFC-76-NH_2_/Cl-67% (Figures S20, S21 and S29 in SI).

Despite the similar multivariate ratio, these MTV samples show substantial differences in H_2_O adsorption capacities, highlighting the critical role of functional groups in water harvesting. PFC-76-Cl-*y* series exhibited reversible water adsorption isotherms without significant hysteresis behavior (Figures S38–S39 in SI), albeit with a markedly reduced H_2_O uptake capacity. The H_2_O adsorption step of PFC-76-NH_2_/Cl-67% (Figure S39 in SI) closely resembles that of PFC-76-Cl (at *P*/*P*_0_ ∼ 0.6), while its overall H_2_O uptake is comparable to that of PFC-76-NH_2_, which can be attributed to the coexistence of hydrophobic and hydrophilic groups within a confined microenvironment. These findings underscore the significant role of the -NH_2_ functional group as adsorption sites in the H_2_O adsorption process.

Static H_2_O adsorption curve of PFC-76-NH_2_-67% demonstrated a working capacity of 0.14 g g^–1^, calculated under 90% RH with adsorption at 25 °C and desorption at 40 °C (yellow region on Figure 5d). Dynamic vapor sorption (DVS) was used to monitor the dehydration process under different temperatures (Figure 5e, Section S5 in SI). PFC-76-NH_2_-67% absorbs water at 25 °C and gradually reaches equilibrium, with a water uptake of 0.09 g g^–1^ under dynamic conditions, whereas static adsorption achieves a higher uptake of 0.12 g g^–1^ (Figure 5d), attributable to the difference in experimental conditions. In the subsequent desorption process, the water loss reached about 84 wt % at 40 °C and increased further to 89 wt % at 80 °C, indicating less energy cost to regenerate. Additionally, the stability of PFC-76-NH_2_-67% only marginally decreased throughout 50 adsorption–desorption cycles between 0% and 90% RH at 40 °C (Figure 5f, Figure S49 in the SI). After 50 cycles, PFC-76-NH_2_-67% retained 91% of its water capture capacity, and the following PXRD measurements confirmed the high-quality crystallite upon cycling water exposure (Figure S5 in the SI).

Based on the above information, we proposed that water molecules are initially attracted to hydrophilic sites (amino and carboxyl functional groups) through weak hydrogen bonds. With increasing progress, the adsorption mechanism involves both the formation of water clusters and hydrogen bonding between water guests and amino groups of frameworks. In desorption proceeds, the weak hydrogen bonding enables facile release of water molecules, including those initially anchored to amino functionalities. This accounts for the high water desorption efficiency even with a slight increase in temperature.

## Conclusion

In summary, we designed and synthesized a series of isostructural porous MTV-HOFs, where the water adsorption performance and structural stability can be finely tuned by varying the functional groups and their ratios through an MTV strategy. Among these, PFC-76-NH_2_ stands out from other analogs, exhibiting a dynamic structural transformation during both dehydration and hydration processes. SCXRD studies and DFT calculations reveal that the incorporated -NH_2_ groups serve as adsorption sites, facilitating the formation of water clusters between guest molecules and host frameworks via hydrogen bonds. Furthermore, by precise control of the ratio of functional groups, a balance is achieved between the number of adsorption sites and the available pore space. This results in optimized performance for PFC-76-NH_2_-67%, which is characterized by high water uptake, a suitable inflection point in the adsorption step, a low energy barrier for water release, and excellent long-term recyclability. This work not only introduces a novel strategy for developing multifunctional HOF-based materials but also positions MTV-HOFs as a promising platform for water harvesting.

## Supplementary Material











## Notes

Synthetic details, single-crystal structural analysis, static gas/vapor adsorption isotherms, theoretical calculation, ^1^H NMR spectroscopy, FT-IR, PXRD, EDS, contact angles, and DVS (PDF)
Single crystal structure of PFC-76-NH_2_-as (CIF)
Single crystal structure of PFC-76-NH_2_ (CIF)
Single crystal structure of PFC-76-NH_2_-H_2_O (CIF)
Single crystal structure of PFC-76-Cl (CIF)
