# Supplementary material for: Multivariate Hydrogen-Bonded Organic Frameworks for Optimum Atmospheric Water Harvesting
Source: ACS Cent Sci. 2025 Sep 4;11(10):1984–92. doi: 10.1021/acscentsci.5c01233 (PMC12550616; doi:10.1021/acscentsci.5c01233)
Supplement: Supplementary file 1 [file oc5c01233_si_001.pdf]

Supporting Information (67 pages)

Multivariate Hydrogen-Bonded Organic Frameworks for Optimum Atmospheric Water Harvesting

Shan Liu,[§,¶] Lan Li,[†,#,¶] Xiang-Yu Gao,[†,§,¶] Rong Cao,[†,‡] Yue-Biao Zhang,[§,*] and Tian-Fu Liu[†,‡,*]

[†]*State Key Laboratory of Structural Chemistry, Fujian Institute of Research on the Structure of Matter, Chinese Academy of Sciences, Fuzhou 350002, China.*

[§]*School of Physical Science and Technology, Shanghai Key Laboratory of High-Resolution Electron Microscopy, ShanghaiTech University, State Key Laboratory of Advanced Medical Materials and Devices, ShanghaiTech University, Shanghai 201210, China.*

[‡]*University of Chinese Academy of Sciences, Beijing 100049, China.*

[#]*College of Materials and Chemistry, China Jiliang University, Hangzhou 310018, China.*

**Corresponding authors: zhangyb@shanghaitech.edu.cn and tfliu@fjirsm.ac.cn*

Table of Contents

Section S1. Materials Preparation and Characterization 3

Section S2. [1]H Nuclear Magnetic Resonance Spectroscopy 16

Section S3. Gas Adsorption Isotherms 24

Section S4. Single-Crystal X-Ray Crystallography 34

Section S5. H_2O adsorption and Harvest 42

Section S6. Theorical Calculation and Molecular Simulation 53

References 67

Section S1. Materials Preparation and Characterization

Materials. Unless otherwise mentioned, all reagents and solvents were purchased from commercial sources and used as received without further purification.

Instrumentation. ^{1}H NMR spectra were recorded on Bruker AVANCE III 400MHz spectrometers. Single crystal X-ray diffraction (SCXRD) data was collected at 100 K on a Bruker D8 Venture diffractometer equipped with Cu-Kα radiation (λ = 1.5418 Å). Power X-ray diffraction (PXRD) pattern was performed on Rikagu Miniflex 600 Benchtop X-ray diffraction instrument and Bruker D8 advance diffractometer. Energy dispersive spectrometer (EDS) was performed on Phenom Prox and JSM-IT500HR/LA Prime scanning electron microscope. Contact angle was performed on a KRUSS DSA25 and Biolin Scientific Theta Lite. The CO_2 gas isotherms of the samples were measured using ASAP 2020 from Micromeritics Co. Ltd and BELSOP-MaxII adsorption apparatus. The H_2O gas isotherms of the samples were measured using BelSorp aqua3 from MicroralBEL and Belsorp MAXII from MicroralBEL. Recyclability was tested by DVS Adventure from Surface Measurement Systems.

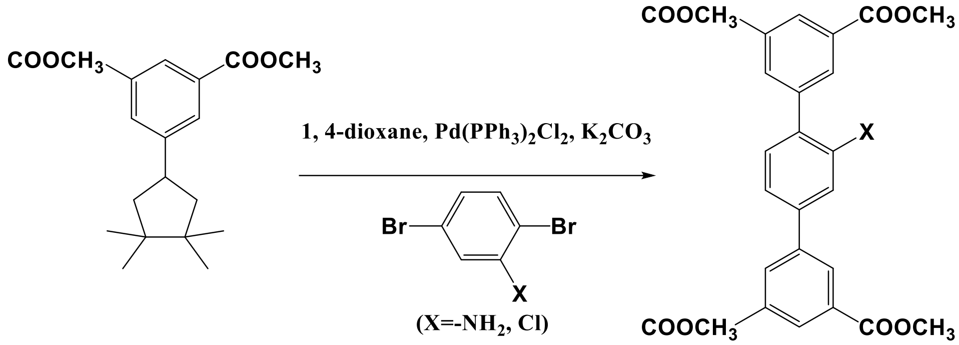

Scheme S1. Synthesis of $(CH_3)_4$TPTCA-X.

Synthesis of $(CH_3)_4$TPTCA-X (X = H, NH_2, Cl).[1] Mixture of dimethyl 5-(pinacolboryl) isophthalate (1.92 g, 6 mmol), 2,5-dibromoaniline (0.5 g, 2 mmol) or 1,4-dibromo-2-chlorobenzene (0.54 g, 2mmol), 2.78 g K_2CO_3 (2.78 g, 20 mmol) in dry dioxane (100 mL) was stirred under the protection of N_2 for 1 h, and then $Pd(PPh_3)_2Cl_2$ (60 mg, 0.06 mmol) was put into system under the increased nitrogen flow. The reaction was carried out at 85 °C for 72 h. The mixture was put into water (100 mL) and extracted by chloroform (200 mL) for three times. The organic phase was dried with magnesium sulfate and the solvent was removed under vacuum. The product was obtained after recrystallization in toluene (yield = 70%).

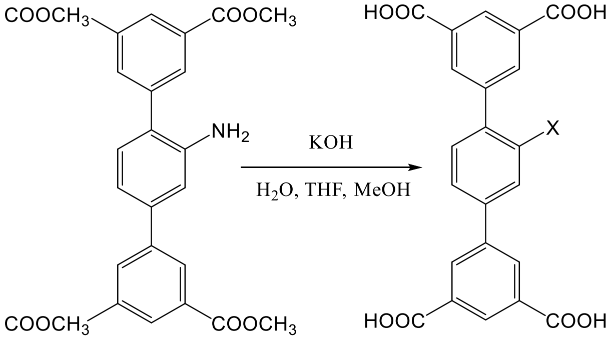

Scheme S2. Synthesis of H_4TPTCA-NH_2

Synthesis of H_4TPTCA-X. 0.4 g (17.8 mmol, 12 equiv) KOH was added to the suspension of $(CH_3)_4$TPTCA-X (0.4 g ,0.823 mmol) in 40 mL of THF/MeOH/H_2O (v/v = 5:2:2), and the mixture was stirred under reflux at 85 °C for 12 h. The solvent was removed under vacuum, and then 100 mL H_2O was added to the residue. The mixture (yellow clear solution) was stirred at room temperature for 2 h. The pH value was adjusted to 2 using concentrated HCl. The resulting light-yellow solids were collected by filtration and washed with water several times. After drying under vacuum, 0.88 g (1.29 mmol) product was obtained with a yield of 97%.

Preparation of PFC-76 for Single crystal X-ray diffraction analysis. 10 mg H_4TPTCA, 3.5 mL DMF and 2.5 mL CH_3COOH (AcOH) were put into a 20 mL vial, and then the mixture was under ultrasonic for 30 mins to get uniform suspension. The vial was put in 130 °C oven and the cap was unscrewed. The solvent would evaporate slowly and the white needle crystals of TPTCA HOF suitable for single crystal x-ray diffraction analysis were grown, when the solvent is almost dry.

Preparation of PFC-76-NH_2 for Single crystal X-ray diffraction analysis. 10 mg H_4TPTCA-NH_2, 3.5 mL DMF and 2.5 mL AcOH were put into a 20 mL vial, and then the mixture was under ultrasonic for 30 mins to get uniform suspension. The vial was put in 130 °C oven and the cap was unscrewed. The solvent would evaporate slowly and the white needle crystals of TPTCA HOF suitable for single crystal x-ray diffraction analysis were grown, when the solvent is almost dry.

Preparation of PFC-76-Cl for Single crystal X-ray diffraction analysis. 10 mg H_4TPTCA-Cl, 3.5 mL DMF and 2.5 mL AcOH were put into a 20 mL vial, and then the mixture was under ultrasonic for 30 mins to get uniform suspension. The vial was put in 130 °C oven and the cap was unscrewed. The solvent would evaporate slowly and the white needle crystals of TPTCA HOF suitable for single crystal x-ray diffraction analysis were grown, when the solvent is almost dry.

Synthesis of powdery PFC-76 with micron-size. 75 mg H_4TPTCA was added into10 mL AcOH in a 20 mL vial, and the mixture was under ultrasonic for 30 mins to get uniform suspension. The mixture was capped and heated to 90 °C for 12 h.

Synthesis of powdery PFC-76-NH_2 with micron-size. 75 mg H_4TPTCA-NH_2 was added into 10 mL AcOH in a 20 mL vial, and the mixture was under ultrasonic for 30 min to get uniform suspension. The mixture was capped and heated to 90 °C for 12 h.

Synthesis of powdery PFC-76-Cl HOF with micron-size. 75 mg H_4TPTCA-Cl was added into 10 mL AcOH in a 20 mL vial, and the mixture was under in ultrasonic for 30 mins to get uniform suspension. The mixture was capped and heated to 90 °C for 12 h.

Synthesis of powdery MTV PFC-76-NH_2-50% HOF with micron-size. 37.5 mg H_4TPTCA and 37.5 mg H_4TPTCA-NH_2 were added into 10 mL AcOH in a 20 mL vial, and the mixture was under ultrasonic for 30 min to get uniform suspension. The mixture was capped and heated to 90 °C for 12 h.

Synthesis of powdery MTV PFC-76-NH_2-67% HOF with micron-size. 25 mg H_4TPTCA and 50 mg H_4TPTCA-NH_2 were added into 10 mL AcOH in a 20 mL vial, and the mixture was under ultrasonic for 30 min to get uniform suspension. The mixture was capped and heated to 90 °C for 12 h.

Synthesis of powdery MTV PFC-76-NH_2-80% HOF with micron-size. 15 mg H_4TPTCA and 60 mg H_4TPTCA-NH_2 were added into 10 mL AcOH in a 20 mL vial and the mixture was under ultrasonic for 30 min to get uniform suspension. The mixture was capped and heated to 90 °C for 12 h.

Synthesis of powdery MTV PFC-76-Cl-50% HOF with micron-size. 37.5 mg H_4TPTCA and 37.5 mg H_4TPTCA-Cl were added into 10 mL AcOH in a 20 mL vial and the mixture was under ultrasonic for 30 min to get uniform suspension. The mixture was capped and heated to 90 °C for 12 h.

Synthesis of powdery MTV PFC-76-Cl-67% HOF with micron-size. 25 mg H_4TPTCA and 50 mg H_4TPTCA-Cl were added into 10 mL AcOH in a 20 mL vial and the mixture was under ultrasonic for 30 min to get uniform suspension. The mixture was capped and heated to 90 °C for 12 h.

Synthesis of powdery MTV PFC-76-NH_2/Cl-67% HOF with micron-size. 25 mg H_4TPTCA-NH_2 and 50 mg H_4TPTCA-Cl were added into 10 mL AcOH in a 20 mL vial and the mixture was under ultrasonic for 30 min to get uniform suspension. The mixture was capped and heated to 90 °C for 12 h.

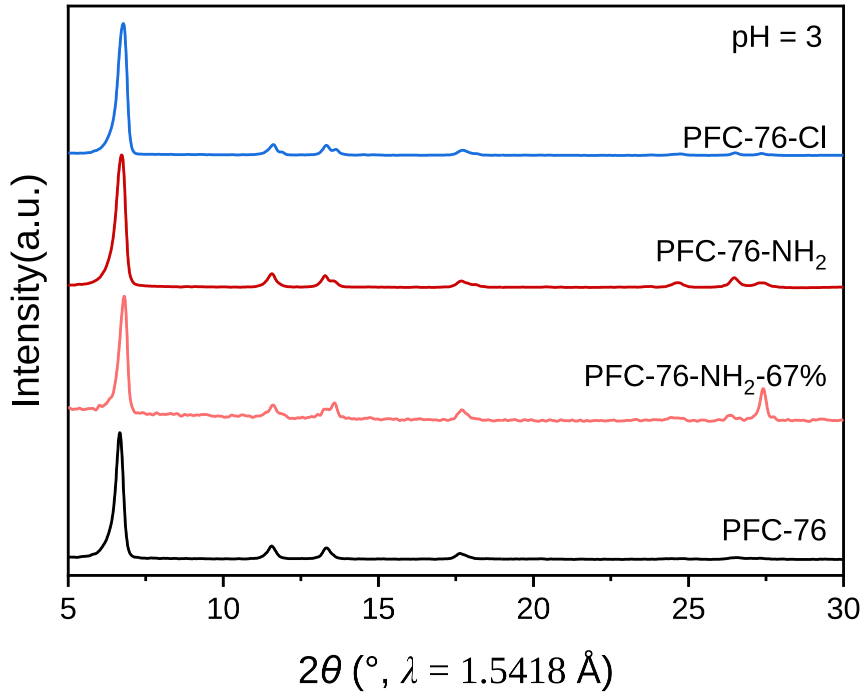

Figure S1. PXRD patterns of PFC-76-X (X = H, NH_2, and Cl) and PFC-76-NH_2-67% sample immersed in pH 3 HCl solution for 2 weeks.

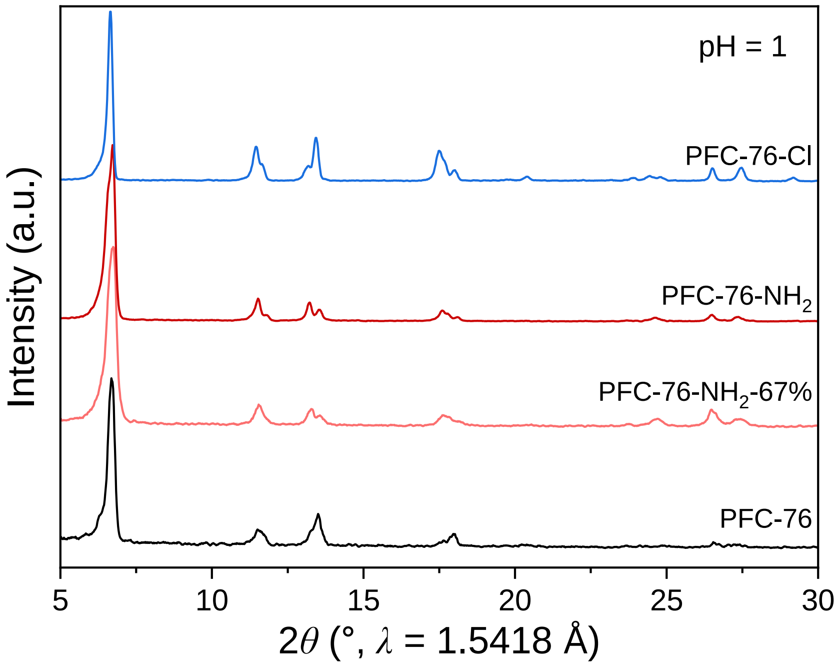

Figure S2. PXRD patterns of PFC-76-X (X = H, NH_2, and Cl) and PFC-76-NH_2-67% sample immersed in pH 1 HCl solution for 2 weeks.

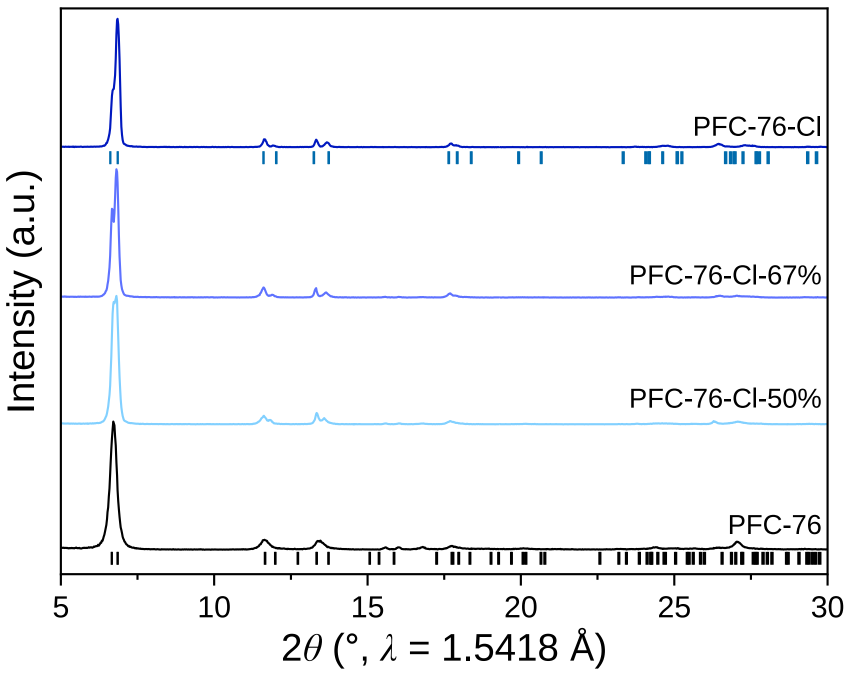

Figure S3. PXRD patterns of PFC-76 and MTV PFC-76-Cl-y (y = 50%, 67% and 100%) samples.

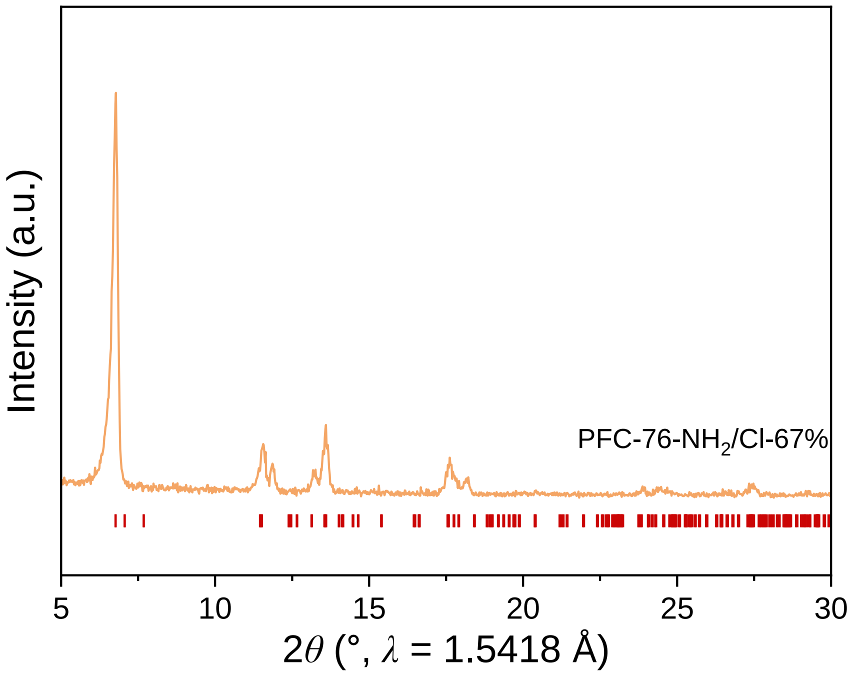

Figure S4. PXRD patterns of MTV PFC-76-NH_2/Cl-67% sample.

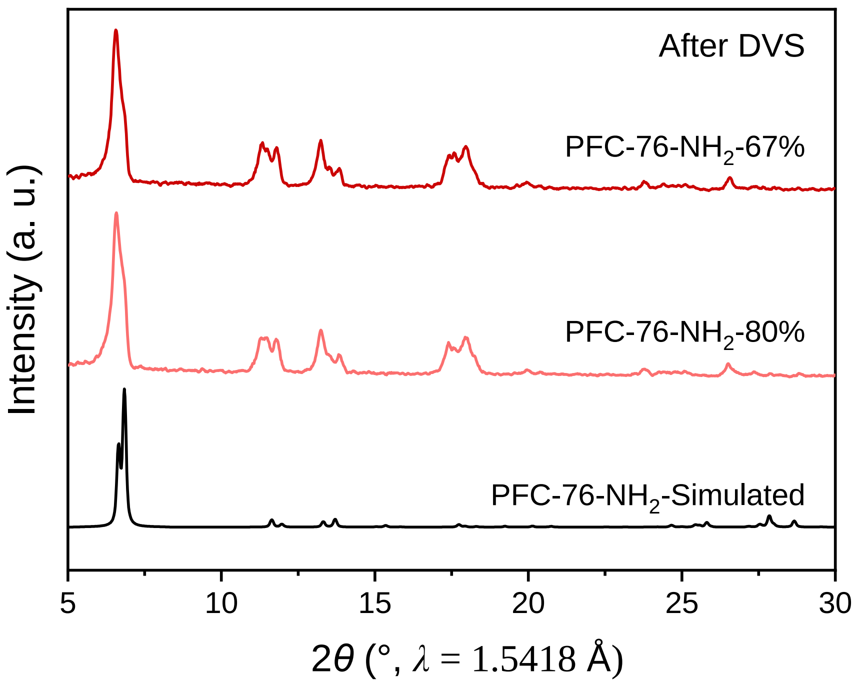

Figure S5. PXRD patterns of PFC-76-NH_2-67% and -80% after DVS.

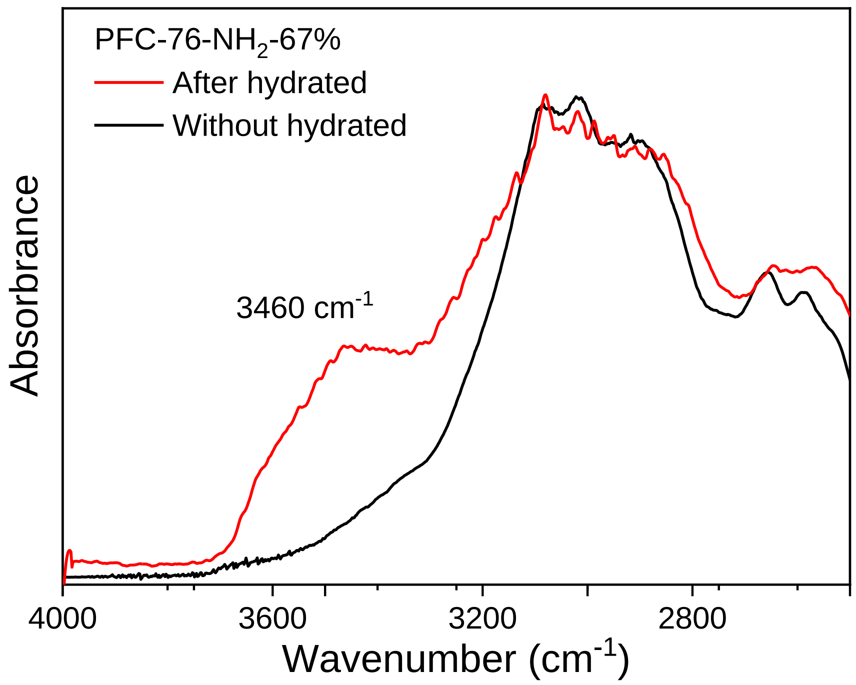

Figure S6. The FT-IR spectra of PFC-76-NH_2-67% after hydrated (Red) and without hydrated (Black).

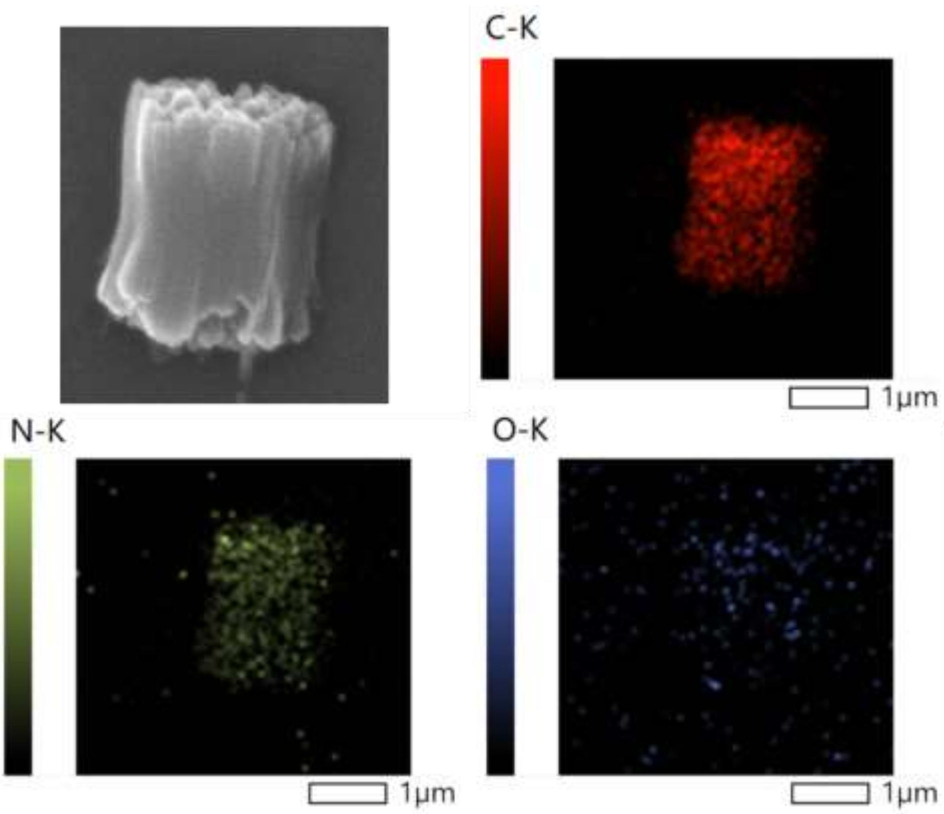

Figure S7. Typical energy dispersive X-ray spectroscopy (EDS) elemental mapping images of PFC-76-NH_2-67%.

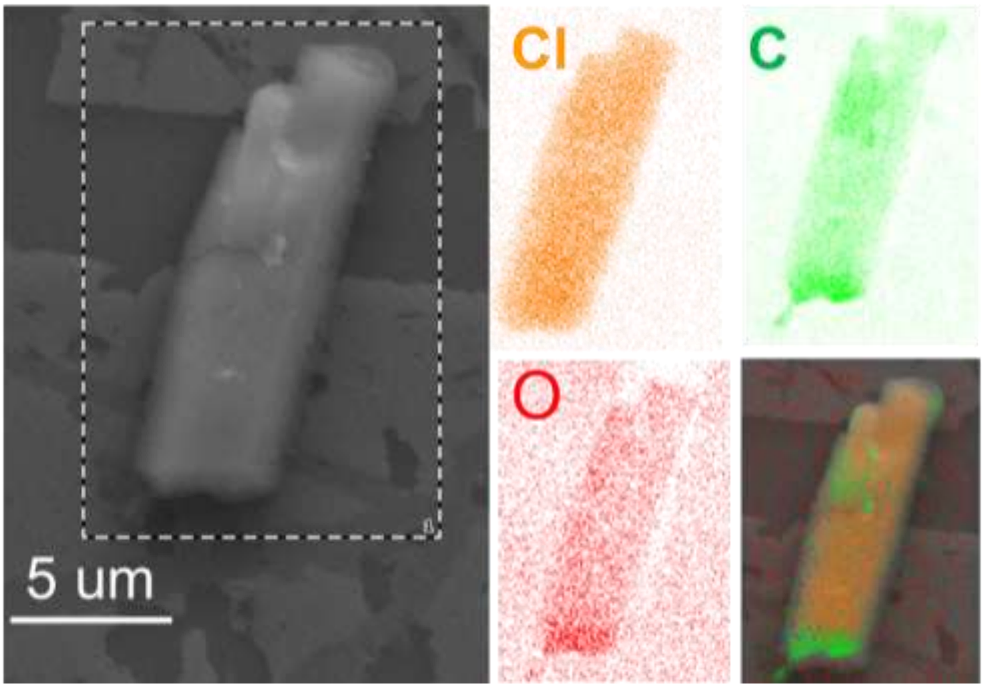

Figure S8. EDS elemental mapping images of PFC-76-Cl-67%

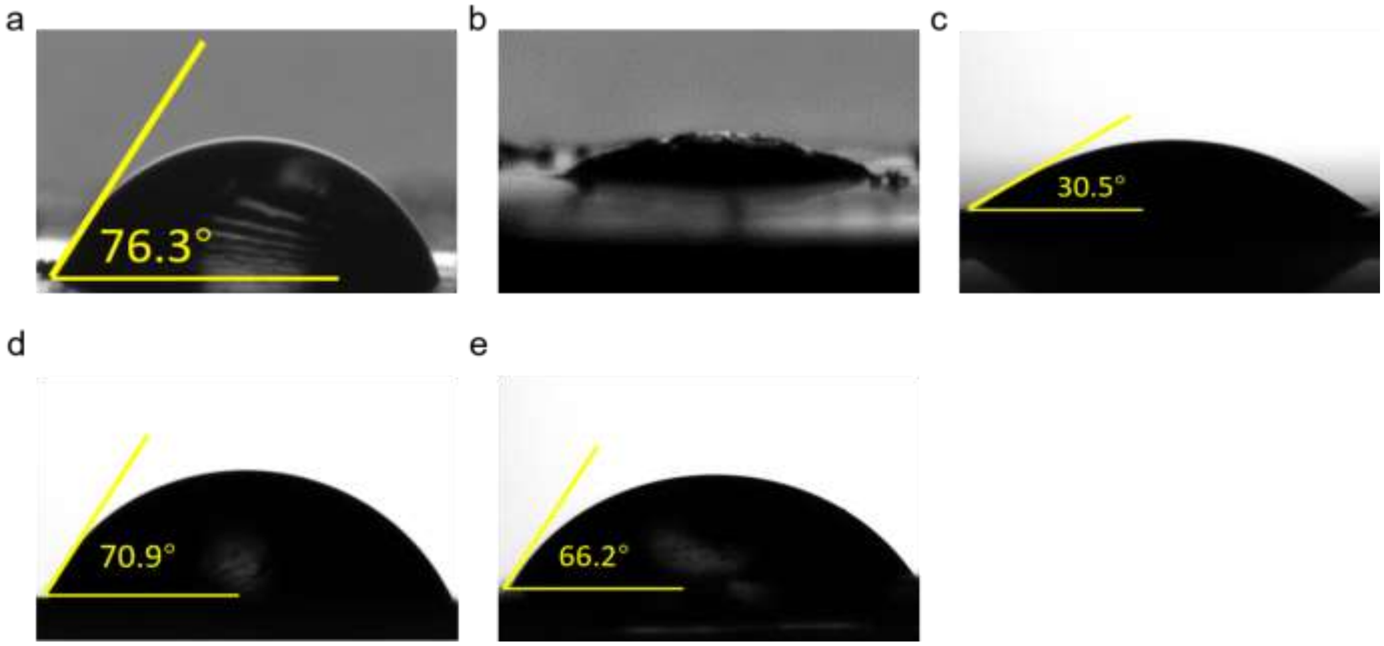

Figure S9. Contact angel of (a) PFC-76, (b) PFC-76-NH_2, (c) PFC-76-NH_2-67%, (d) PFC-76-Cl, (e) PFC-76-Cl-67%.

Section S2. ^{1}H Nuclear Magnetic Resonance Spectroscopy

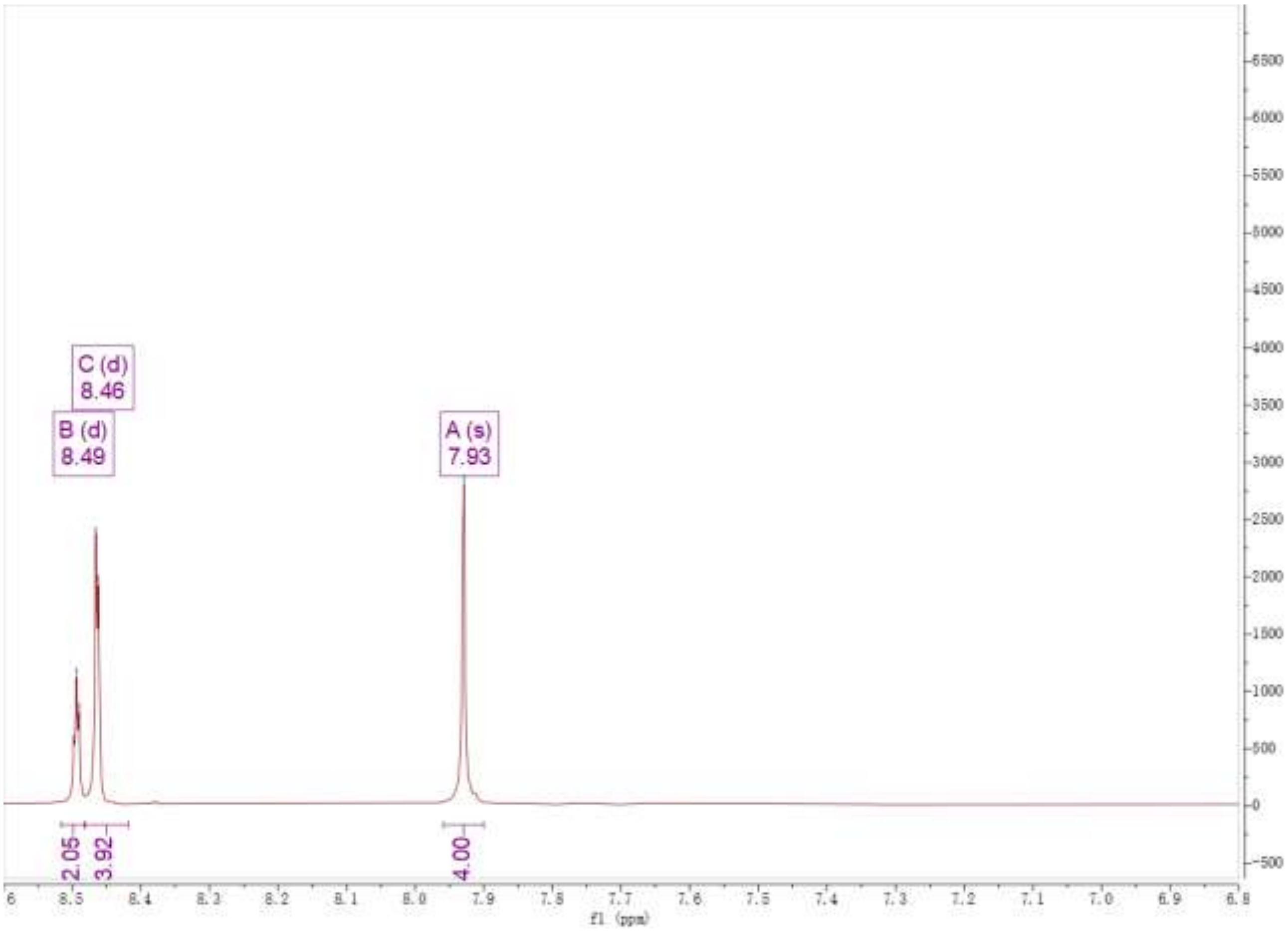

Figure S10. ^{1}H NMR spectroscopy of H_4TPTCA.

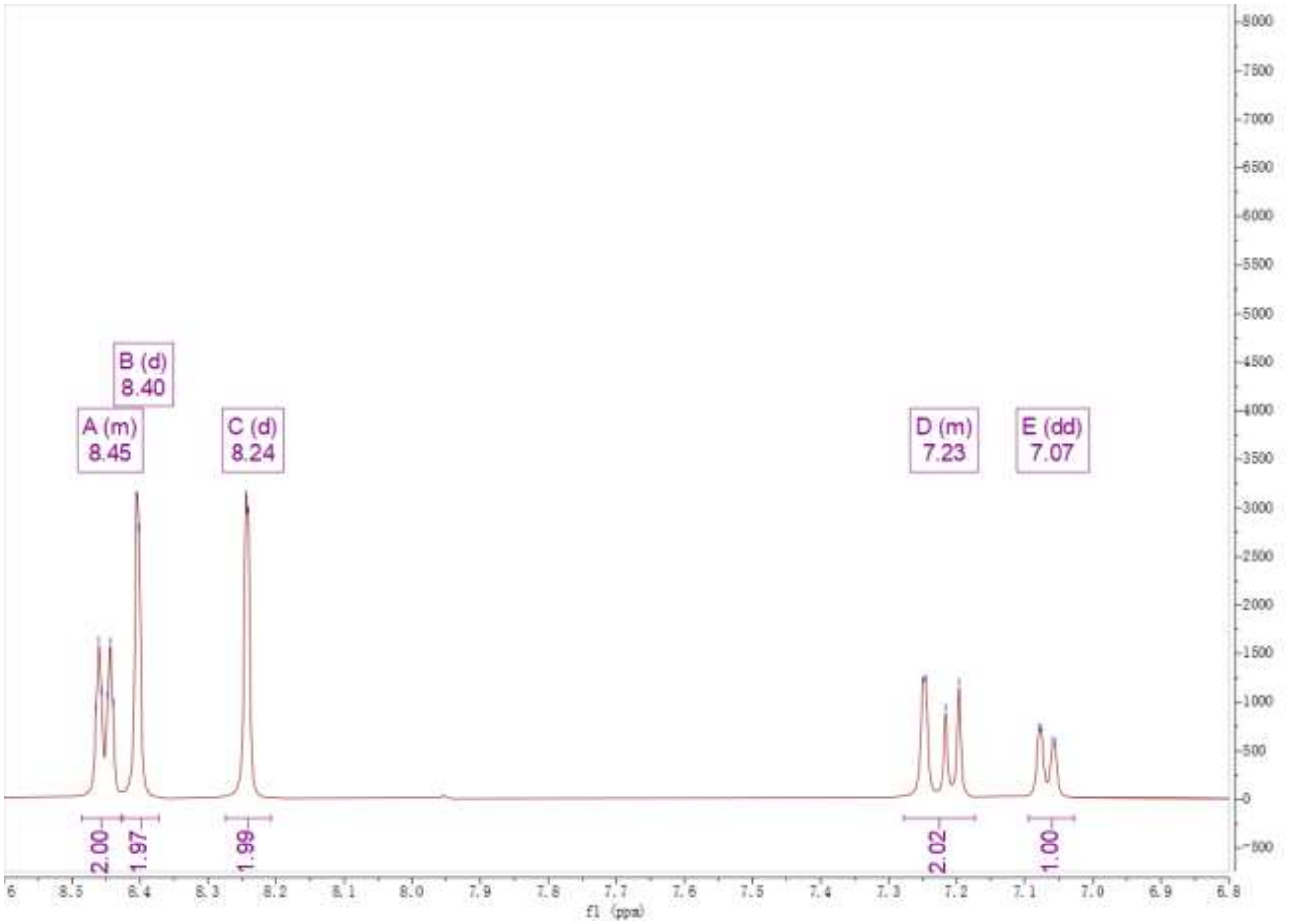

Figure S11. ^{1}H NMR spectroscopy of H_4TPTCA-NH_2.

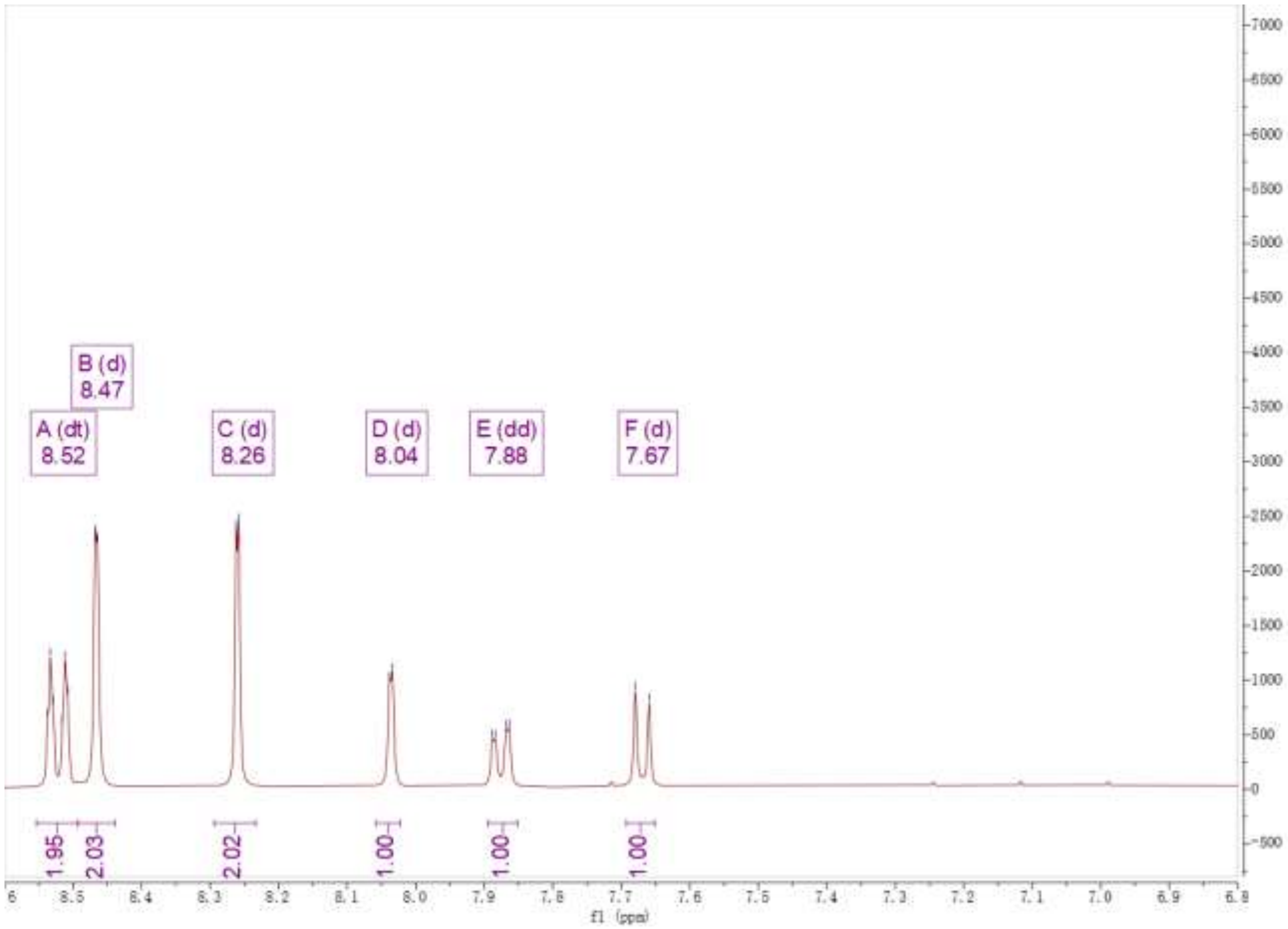

Figure S12. ^{1}H NMR spectroscopy of H_4TPTCA-Cl.

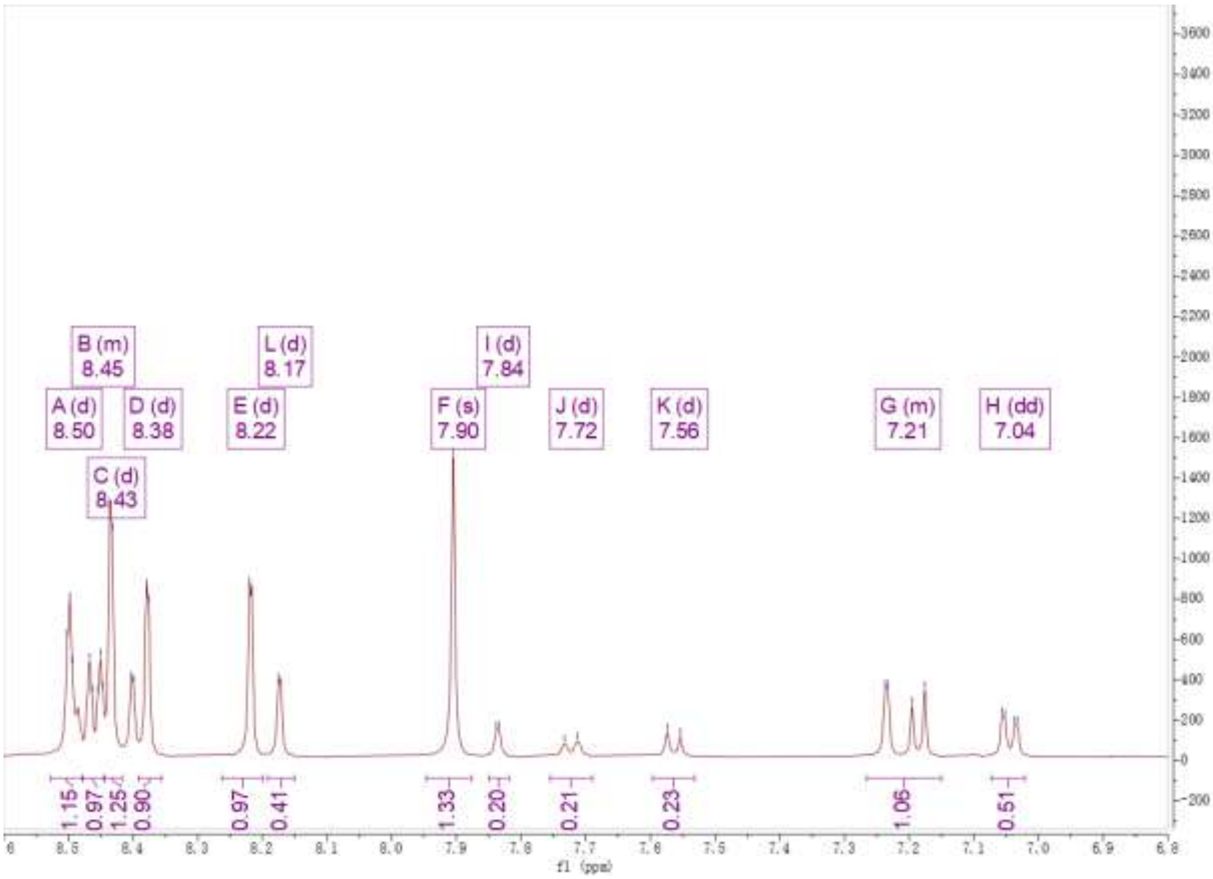

Figure S13. ^{1}H NMR spectroscopy of PFC-76-NH_2-67%.

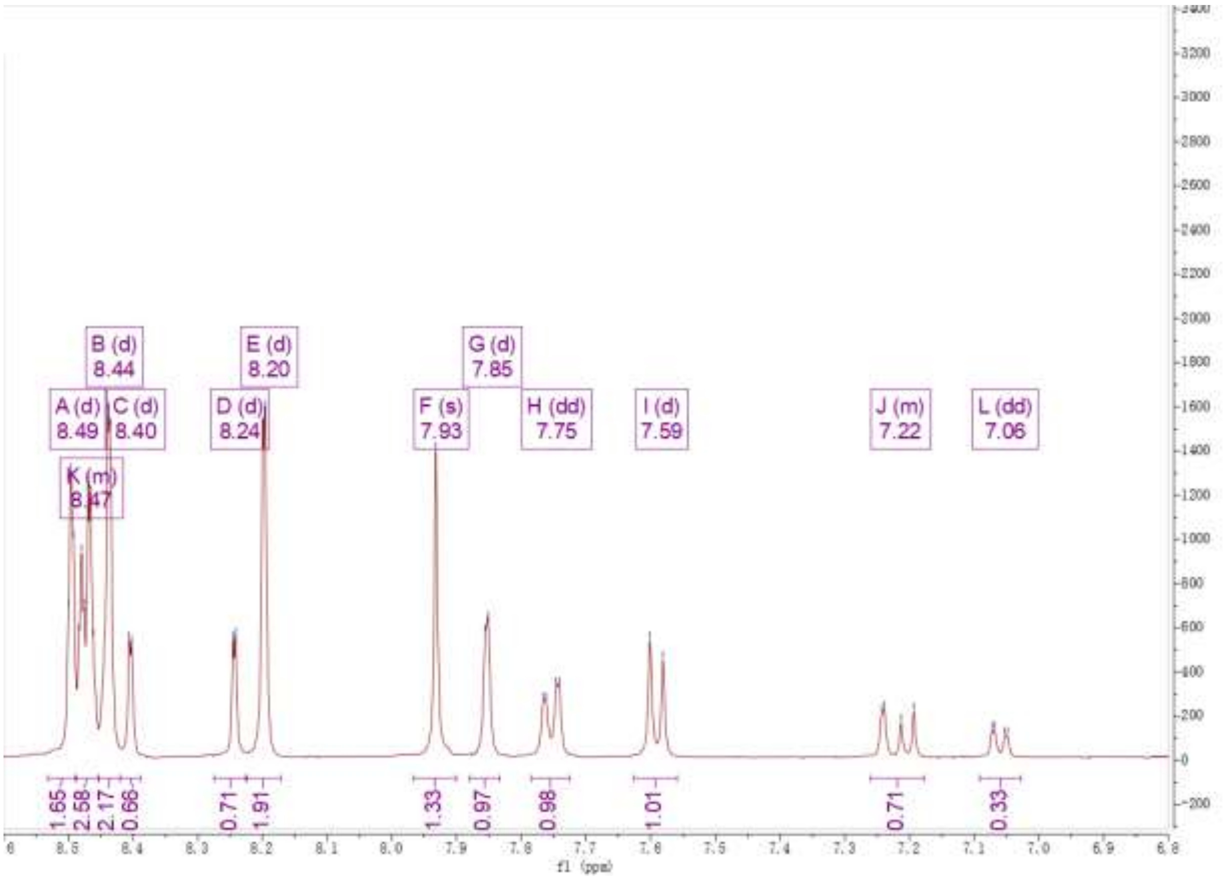

Figure S14. ^{1}H NMR spectroscopy of PFC-76-NH_2-67% immersed in pH 9 NaOH solution for 8 h.

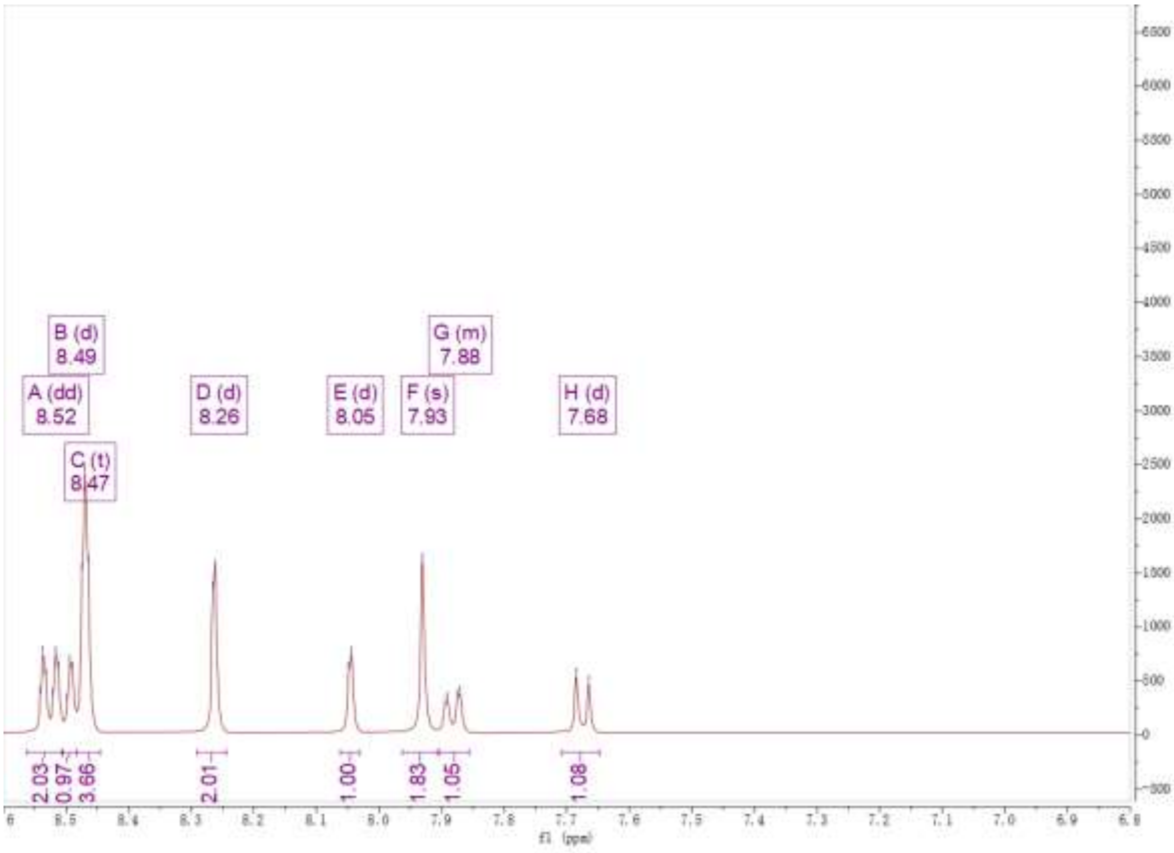

Figure S15. ^{1}H NMR spectroscopy of PFC-76-Cl-67%.

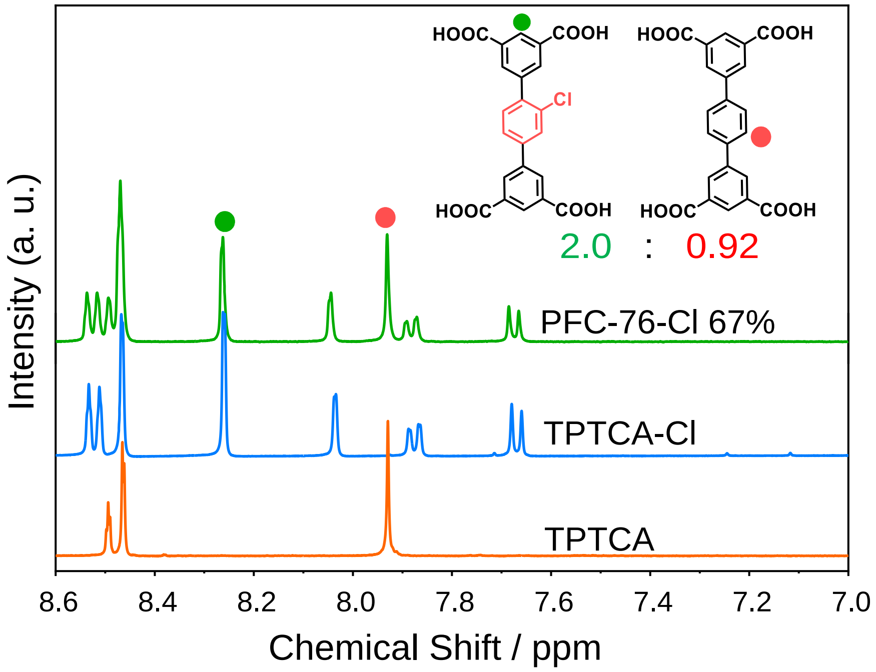

Figure S16. ^{1}H NMR spectroscopy of MTV-PFC-76-Cl-67%.

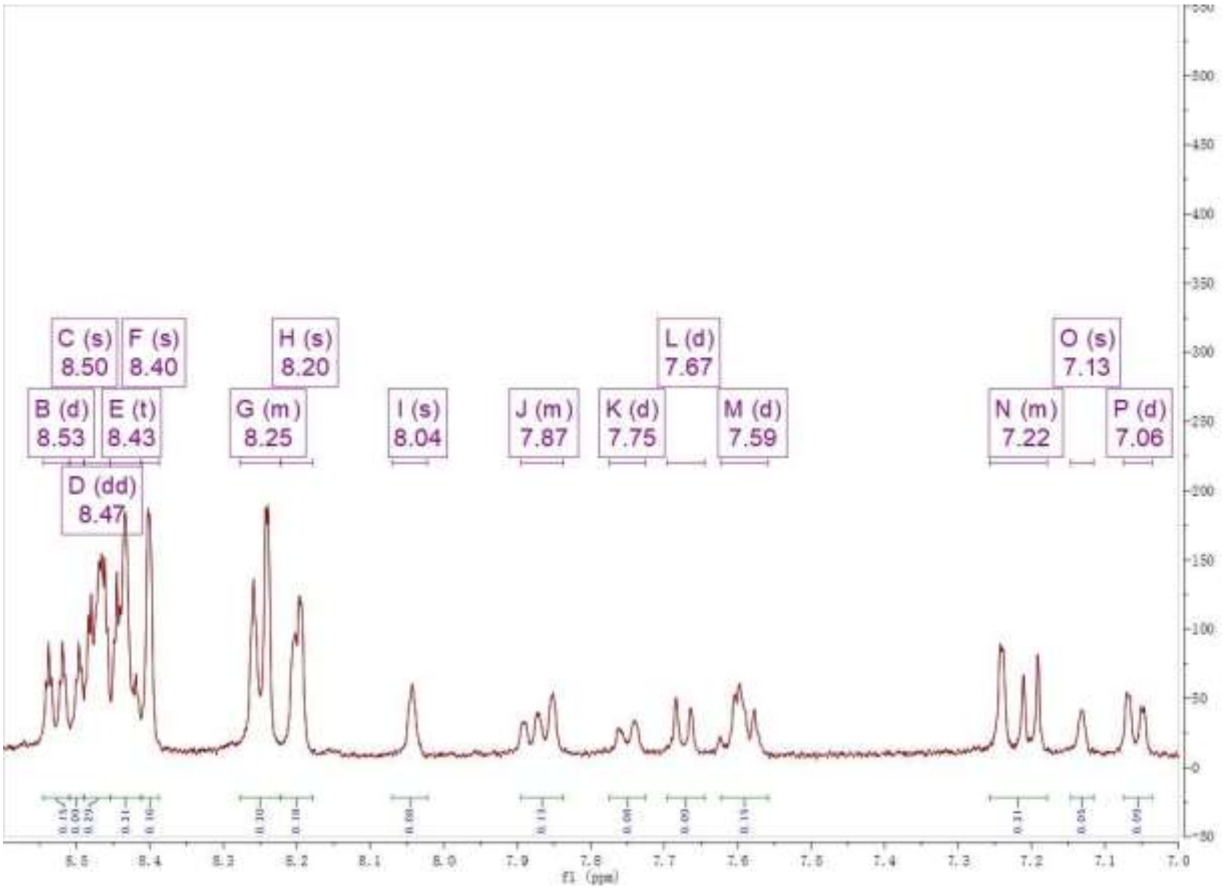

Figure S17. ^{1}H NMR spectroscopy of PFC-76-NH_2/Cl-67%.

Section S3. Gas Adsorption Isotherms

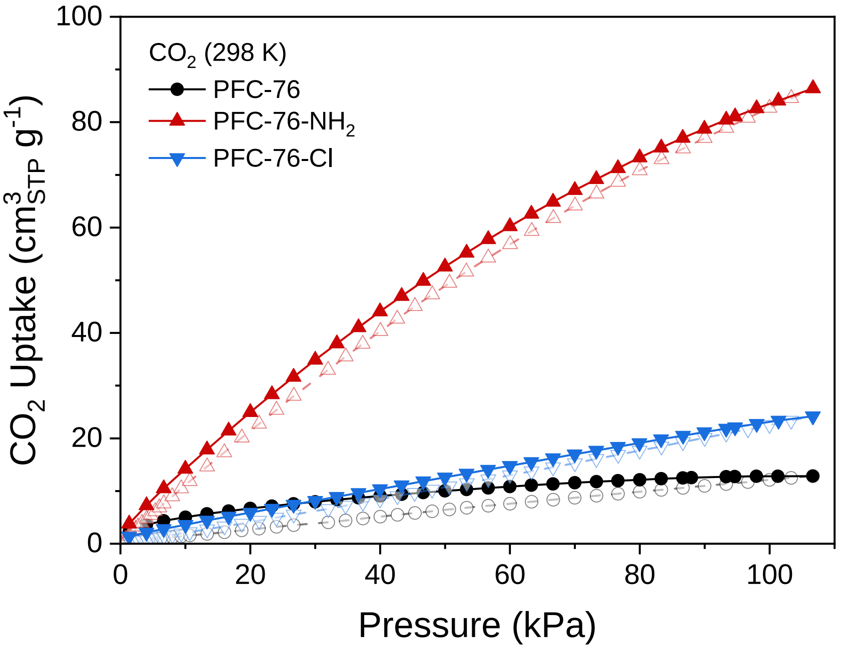

Figure S18. 298 K CO_2 isotherms for a series of PFC-76-X (X = H, NH_2, and Cl) samples.

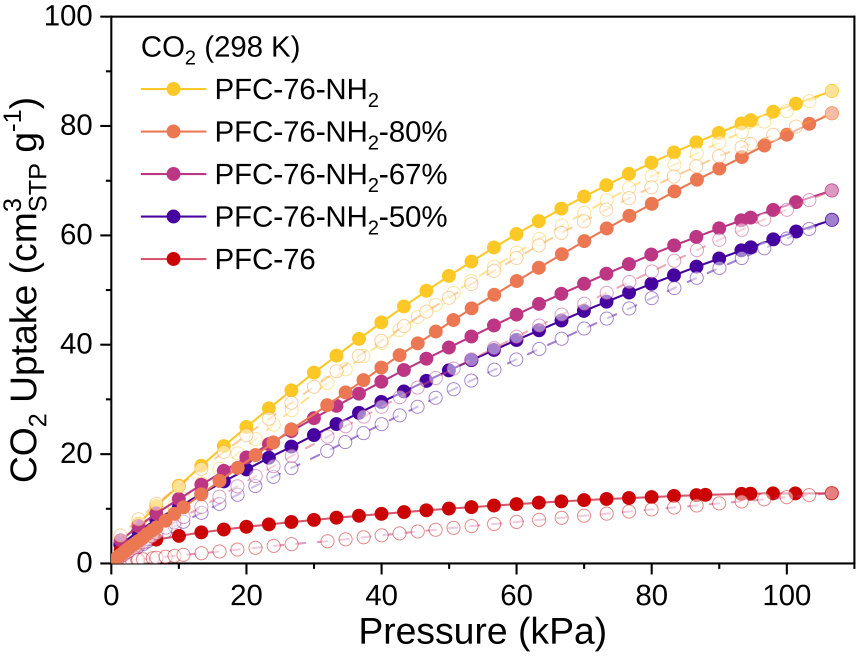

Figure S19. 298 K CO_2 isotherms for a series of MTV PFC-76-NH_2-*x* (*x*=0, 50%, 67%, 80% and 100%) samples.

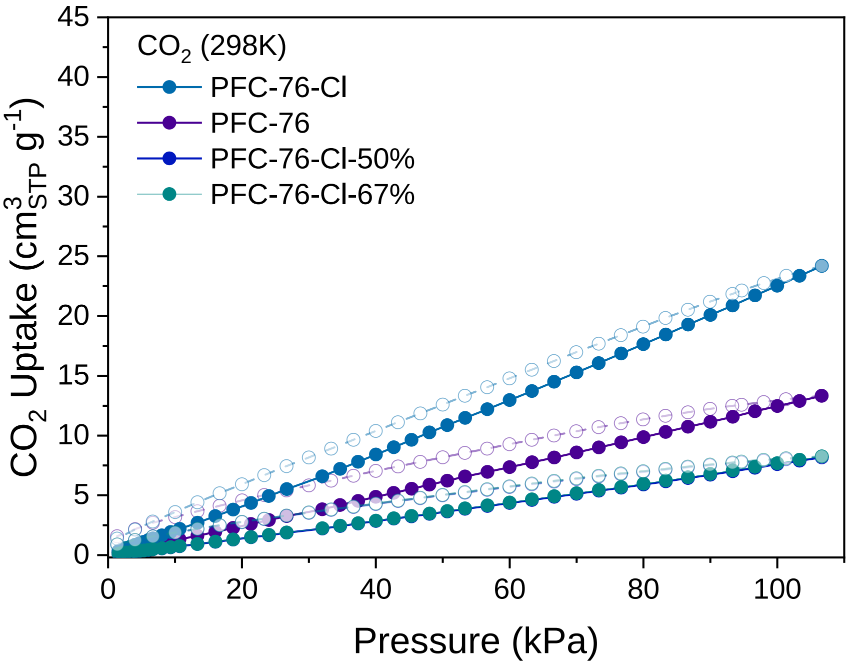

Figure S20. 298 K CO_2 isotherms for a series of MTV PFC-76-Cl-*y* (*y* = 0, 50%, 67%, and 100%) samples.

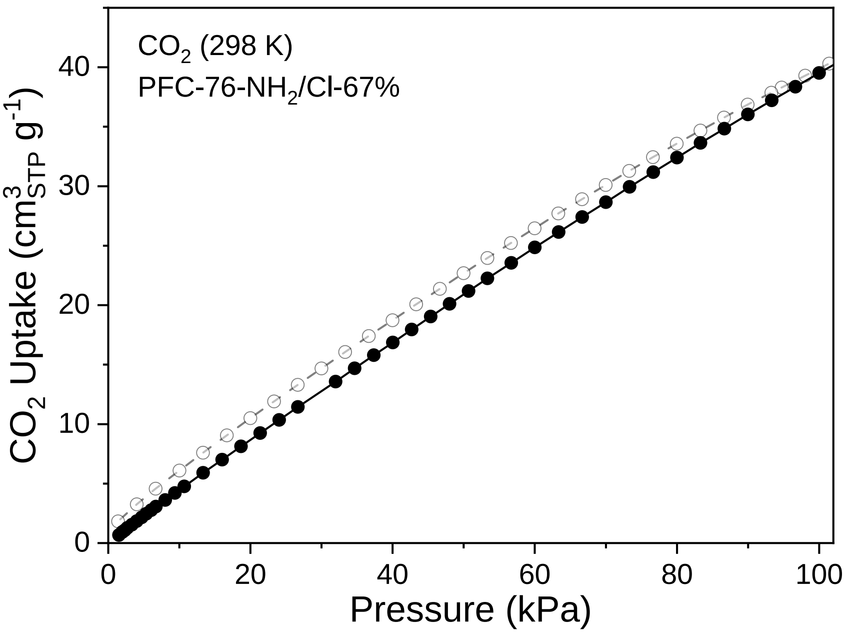

Figure S21. 298 K CO_2 isotherm of MTV PFC-76-NH_2/Cl-67% sample.

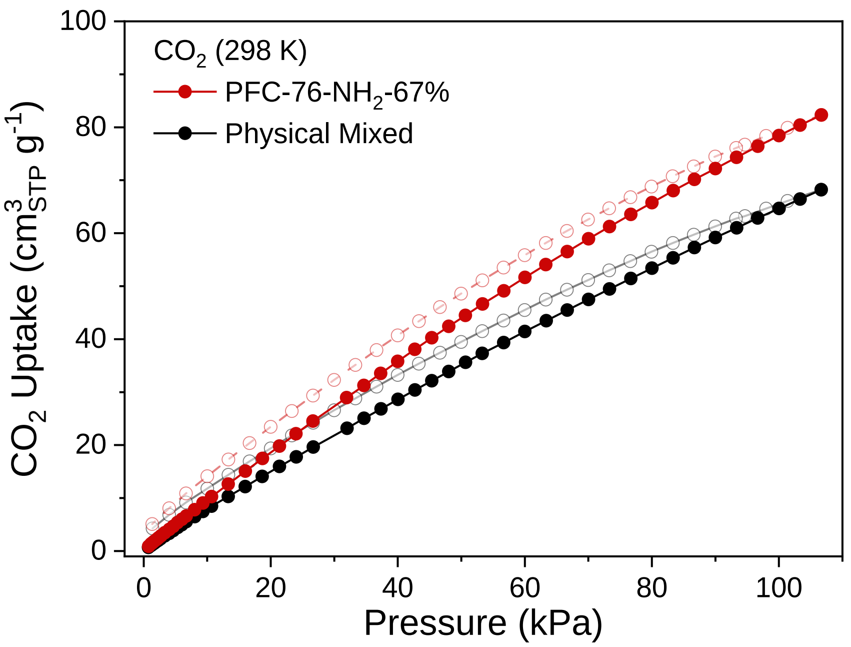

Figure S22. 298 K CO_2 isotherm of PFC-76-NH_2-67% and the sample of physical mixed PFC-76 (33%) and PFC-76-NH_2 (67%).

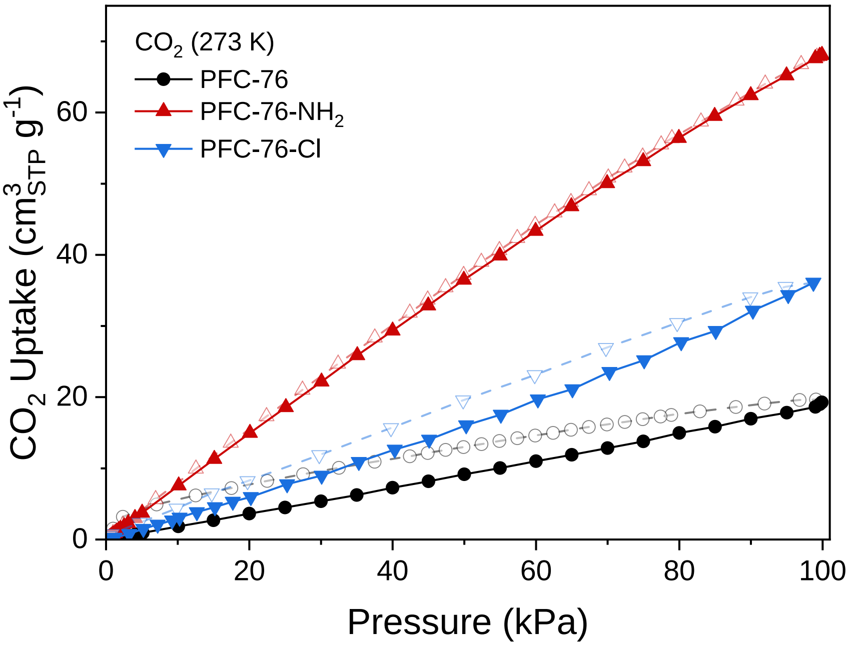

Figure S23. 273 K CO_2 isotherms for a series of PFC-76-X (X = H, NH_2, and Cl) samples.

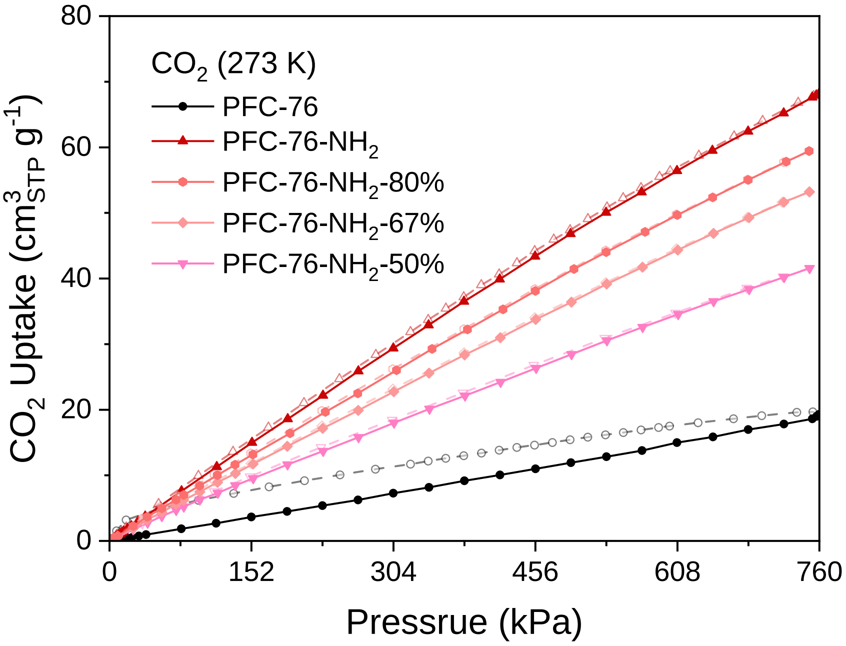

Figure S24. 273 K CO_2 isotherms for a series of MTV PFC-76-NH_2-*x* (*x*=0, 50%, 67%, 80% and 100%) samples.

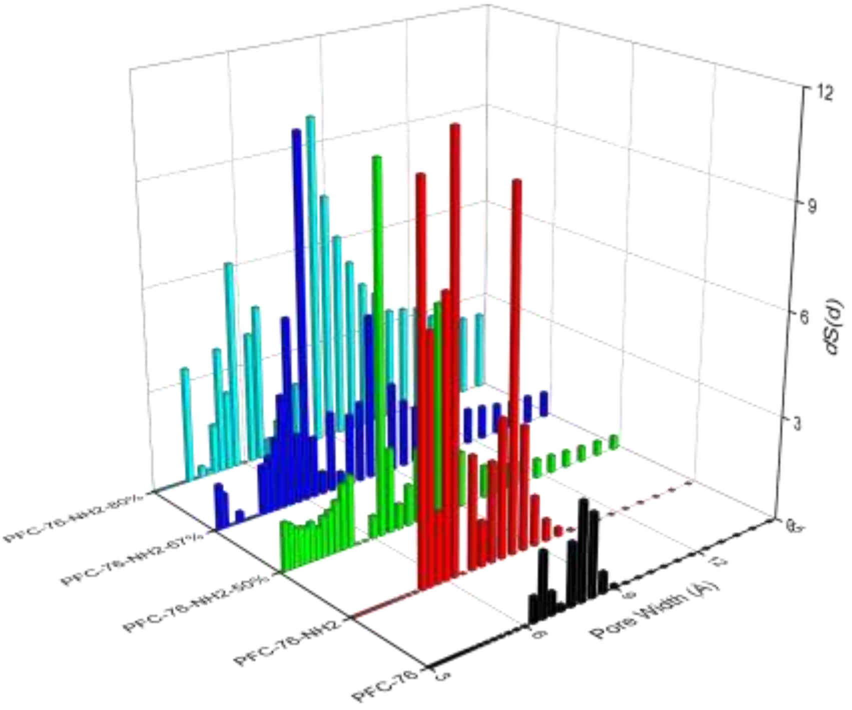

Figure S25. Pore size distribution calculated from the CO_2 adsorption isotherm at 273 K for a series of MTV PFC-76-NH_2-*x* (*x*=0, 50%, 67%, 80% and 100%) samples.

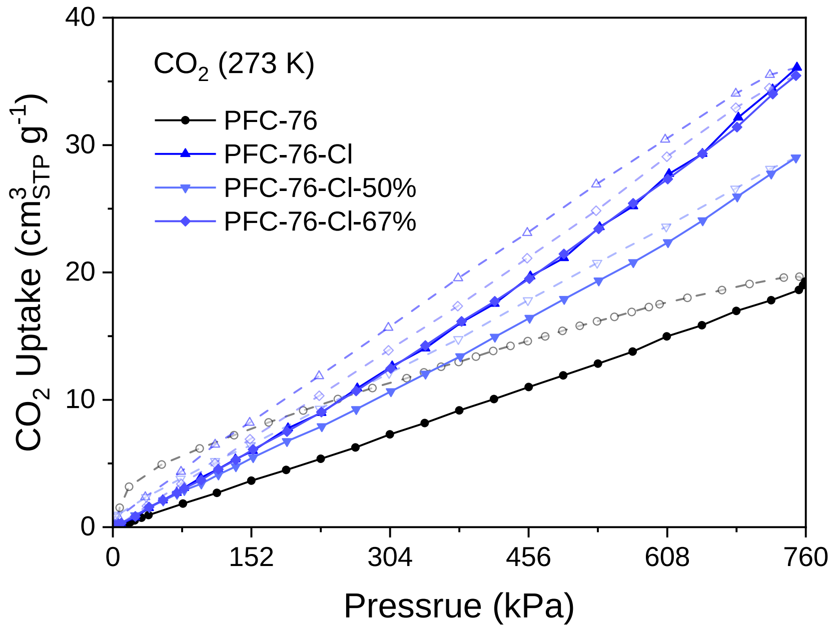

Figure S26. 273 K CO_2 isotherms for a series of MTV PFC-76-Cl-*y* (*y* = 0, 50%, 67%, and 100%) samples.

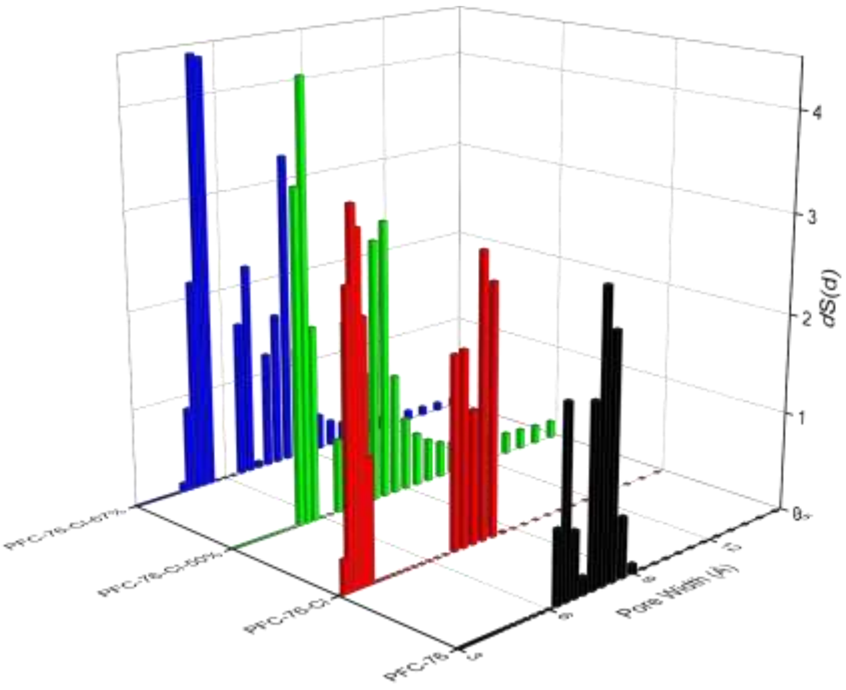

Figure S27. Pore size distribution calculated from the CO_2 adsorption isotherm at 273 K for a series of MTV PFC-76-Cl-*y* (*y* = 0, 50%, 67%, and 100%) samples.

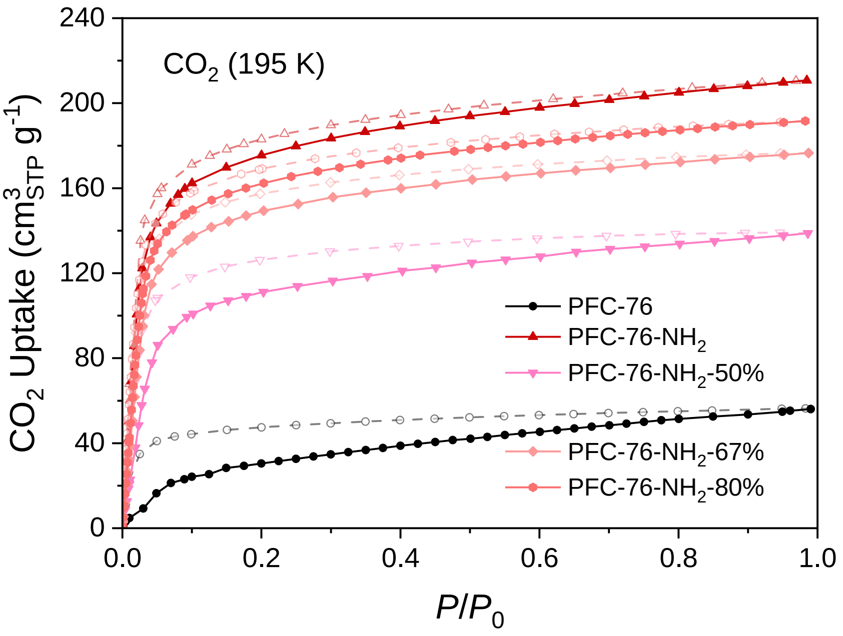

Figure S28. 195 K CO_2 isotherms for a series of MTV PFC-76-NH_2-*x* (*x*=0, 50%, 67%, 80% and 100%) samples.

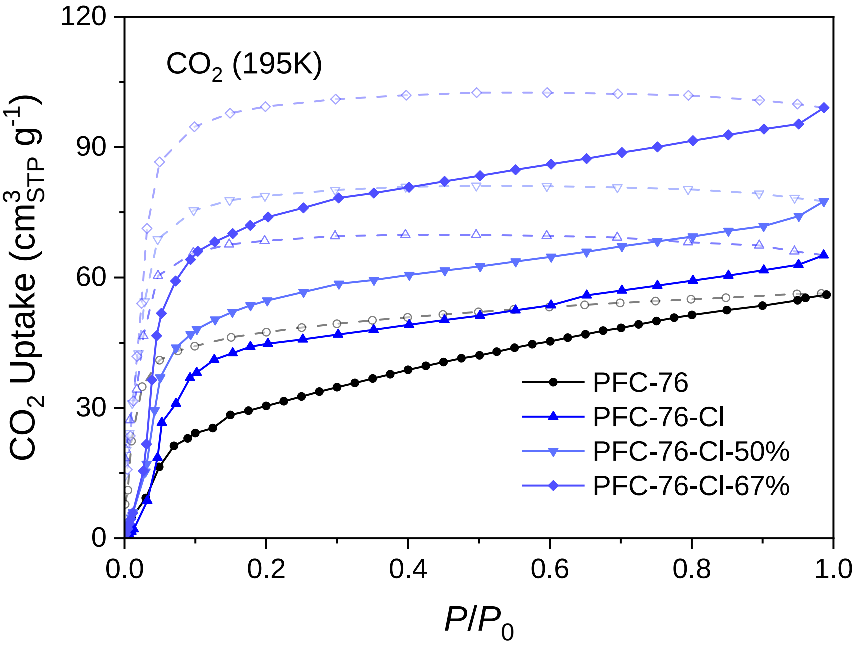

Figure S29. 195 K CO_2 isotherms for a series of MTV PFC-76-Cl-*y* (*y* = 0, 50%, 67%, and 100%) samples.

Section S4. Single-Crystal X-Ray Crystallography

The structure was solved by direct methods using the SHELXTL[2] software package and further refined with different Fourier syntheses. All non-hydrogen atoms on the framework were refined anisotropically. All hydrogen atoms were generated geometrically and refined in the riding mode. All the phenyl rings were treated with rigid constraints.

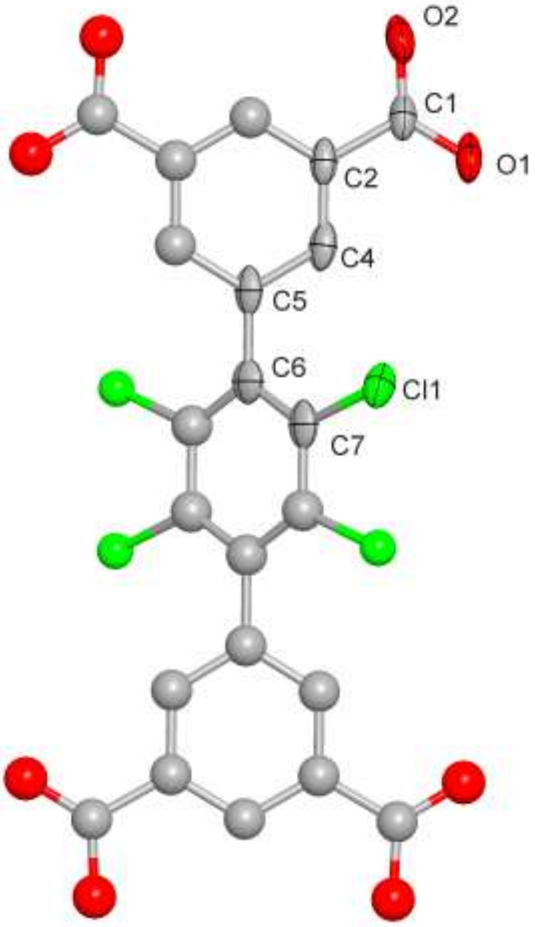

Figure S30. Thermal ellipsoid plot with 50% probability of the asymmetry unit for the single-crystal structure of PFC-76-Cl (C, grey; O, red; Cl, green). Hydrogen atoms are omitted for clarity; Symmetry-related atoms are not labelled and represented as spheres.

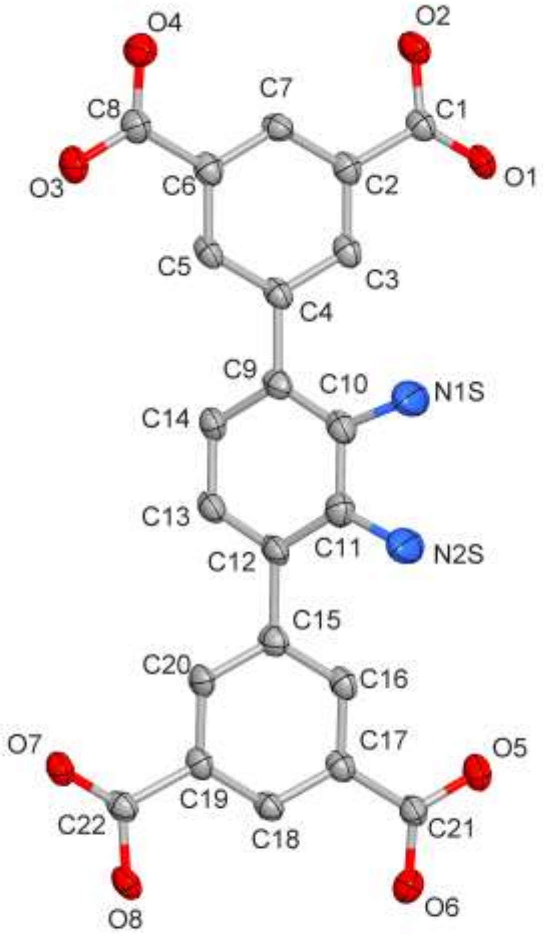

Figure S31. Thermal ellipsoid plot with 50% probability of the asymmetry unit for the single-crystal structure of PFC-76-NH_2-as (C, grey; N, blue; O, red). Hydrogen atoms are omitted for clarity; Symmetry-related atoms are not labelled and represented as spheres.

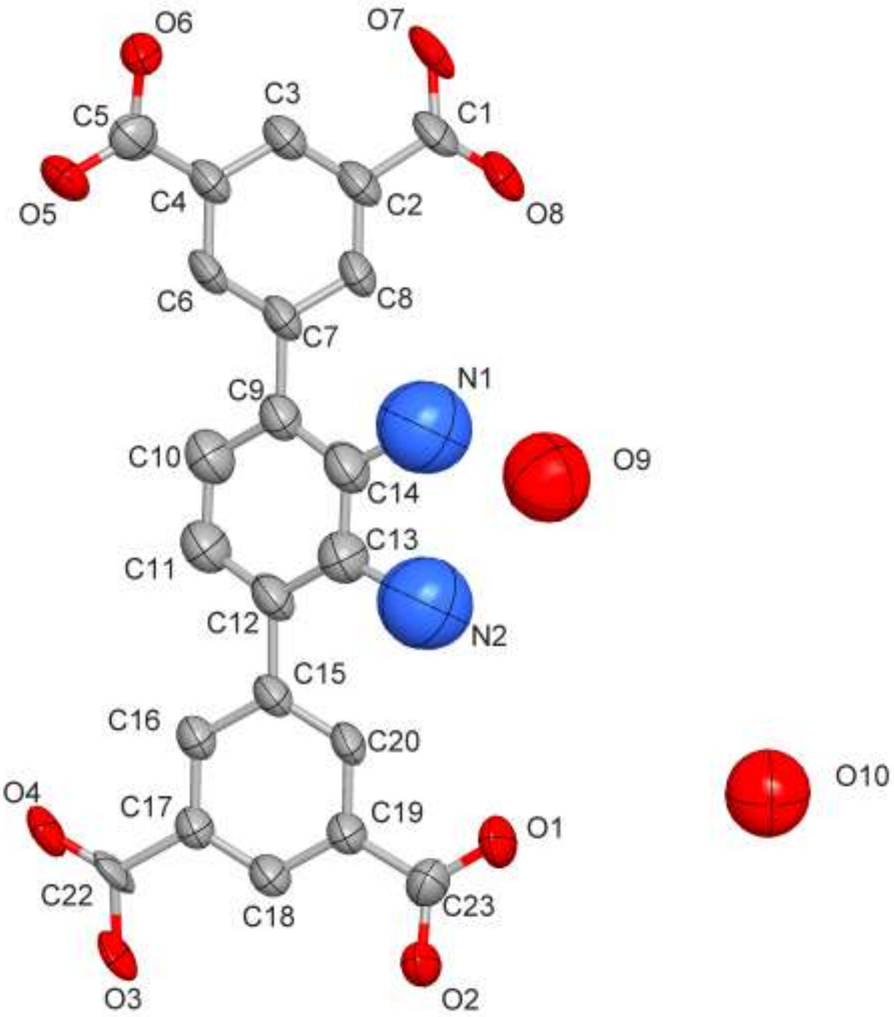

Figure S32. Thermal ellipsoid plot of the asymmetry unit for the single-crystal structure of PFC-76-NH_2 (Framework with 50% probability, water with 25% probability; C, grey; N, blue; O, red). Hydrogen atoms are omitted for clarity; Symmetry-related atoms are not labelled and represented as spheres.

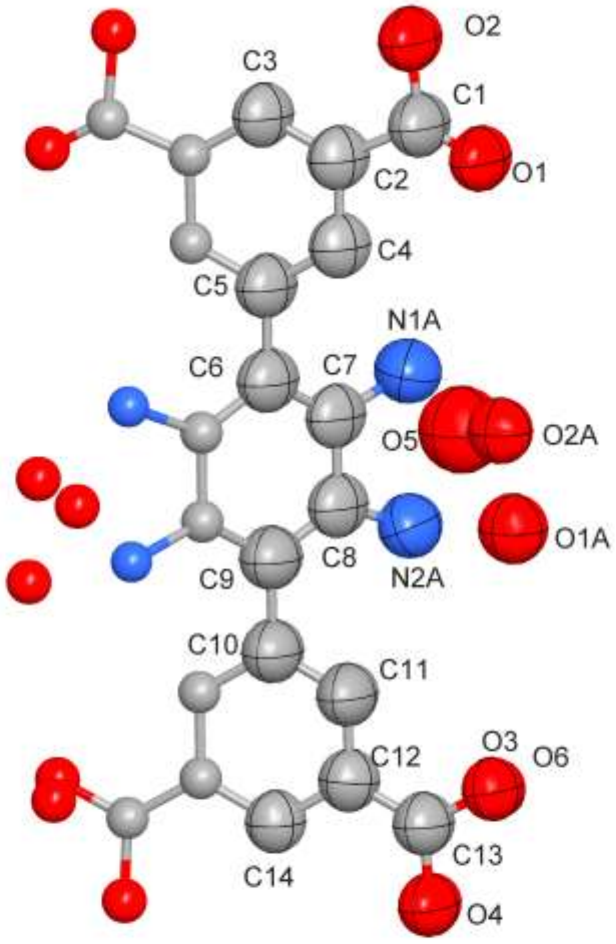

Figure S33. Thermal ellipsoid plot of the asymmetry unit for the single-crystal structure of PFC-76-NH_2-H_2O (Framework with 50% probability, water with 25% probability; C, grey; N, blue; O, red). Hydrogen atoms are omitted for clarity; Symmetry-related atoms are not labelled and represented as spheres.

Table S1. Summary of the crystal data and structure refinement details.

Identification code	PFC-76[3]	PFC-76-Cl	PFC-76-NH_2-as	PFC-76-NH_2	PFC-76-NH_2-H_2O
**Empirical formula**	$C_{22}H_{14}O_8$	$C_{22}H_{10}ClO_8$	$C_{22}H_{15}NO_8$	$C_{44}H_{30}N_2O_{19}$	$C_{22}H_{16}N_2O_{10.3}$
**Formula weight**	406.33	437.75	421.35	890.70	472.65
**Temperature/K**	100.15	150.01	149.99	150.15	293(2)
**Crystal system**	monoclinic	monoclinic	triclinic	triclinic	monoclinic
**Space group**	*C*2/*c*	*C*2/*m*	*P*-1	*P*-1	*I*2/*a*
**a/Å**	16.244(3)	14.736(2)	7.8711(5)	9.181(3)	7.517(3)
**b/Å**	26.568(4)	26.756(4)	13.6765(10)	9.517(4)	26.627(10)
**c/Å**	7.2151(13)	3.6823(5)	14.2066(10)	14.073(5)	14.924(5)
**α/°**	90	90	111.603(3)	99.10(2)	90
**β/°**	114.561(6)	90.767(7)	94.784(3)	98.95(2)	90.14(3)
**γ/°**	90	90	97.385(3)	117.044(19)	90
**V/Å^3**	2832.1(8)	1451.7(4)	1395.86(17)	1044.6(7)	2987.1(18)
**Z**	4	2	2	1	4
**ρ_{calc}g/cm^3**	0.953	1.001	1.002	1.416	1.051
**μ/mm^{-1}**	0.074	0.165	0.078	0.113	0.465
**F(000)**	840	446	436	460	977
**Crystal size/mm^3**	2×1×1	1.0×0.3×0.3	1.0×0.3×0.3	0.8×0.6×0.6	1.0×0.4×0.7
**Radiation**	Mo Kα (λ = 0.71073)				
**2θ range for data collection/°**	5.854–55.178	5.34–45.952	5.272–49.416	5.112–41.632	5.776–49.924
**Index ranges**	-21≤h≤20<br>-34≤k≤27<br>-9≤l≤9	-16≤h≤16<br>-29≤k≤29<br>-4≤l≤4	-9≤h≤9<br>-16≤k≤16<br>-16≤l≤16	-9≤h≤9<br>-9 ≤k≤9<br>-14≤l≤14	-4≤h≤4<br>-14≤k≤26<br>-8≤l≤9
**Reflections collected**	12375	12706	31034	12114	1221
**Independent reflections**	3254	1032	4745	2183	380
**R_{int}**	0.0915	0.0811	0.1051	0.2065	0.4279
**R_{sigma}**	0.0864	0.0353	0.0657	0.1243	0.1864
**Data/restraints/parameters**	3254/0/159	1032/0/79	4745/1/254	2183/179/266	380/265/196
**Goodness-of-fit on F^2**	1.546	1.100	0.994	1.336	1.071
**Final R indexes [I>=2σ (I)]**	R_1 = 0.1315<br>wR_2 = 0.4212	R_1 = 0.0643<br>wR_2 = 0.1936	R_1 = 0.1028<br>wR_2 = 0.2670	R_1 = 0.2387<br>wR_2 =0.5426	R_1 = 0.1782<br>wR_2 = 0.3962
**Final *R* indexes [all data]**	R_1 = 0.2070<br>wR_2 = 0.4704	R_1 = 0.0738<br>wR_2 = 0.2033	R_1 = 0.1271<br>wR_2 = 0.2912	R_1 = 0.3711<br>wR_2 = 0.6304	R_1 = 0.2509<br>wR_2 = 0.4882
**Largest diff. peak/hole / e Å^{-3}**	1.13/-0.43	0.45/-0.23	1.11/-0.44	0.87/-0.68	0.38/-0.48
**CCDC number**	2211272	2449706	2449703	2449704	2449705

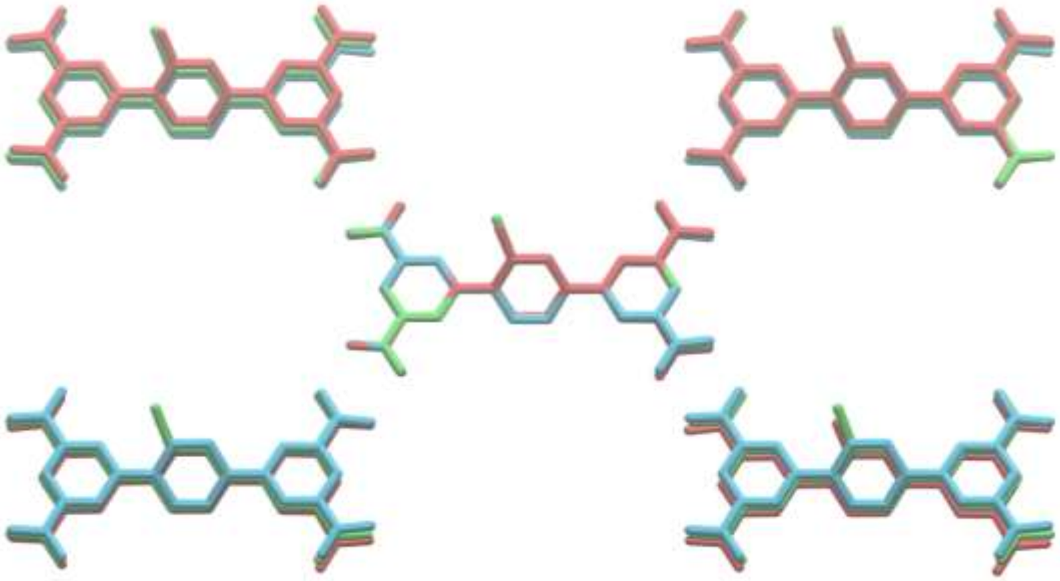

Figure S34. Structural comparison of PFC-76-X (colored with cyan, X = H; red X = NH_2; green X = Cl).

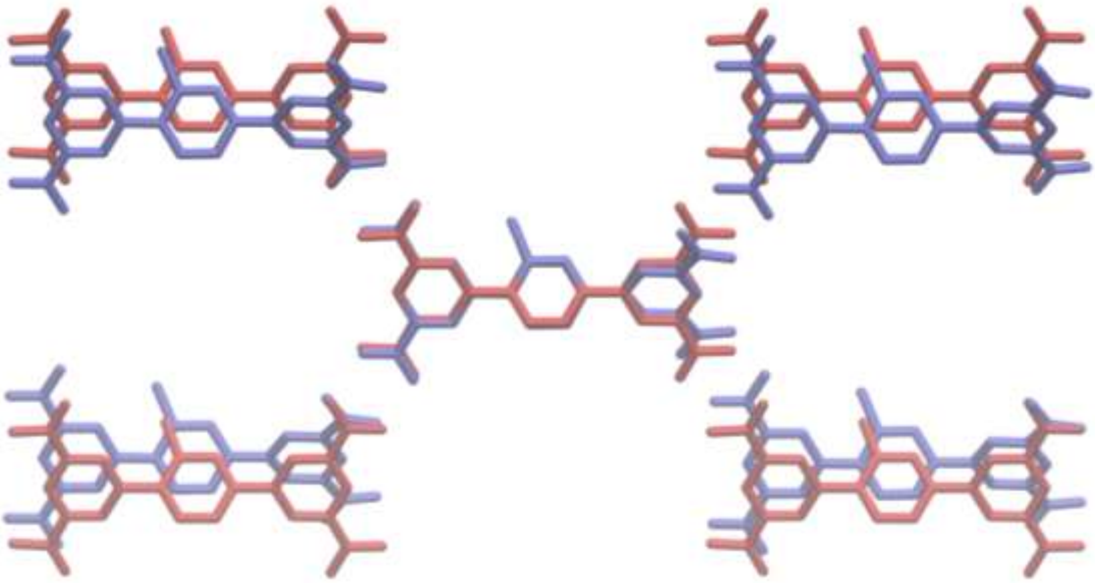

Figure S35. Structural comparison of PFC-76-NH_2 different phase (colored with red, act phase; purple, hydrated phase).

Section S5. H_2O adsorption and Harvest

Organic vapor adsorption isotherms. The organic vapor adsorption isotherms were collected using BELSORP-max and MicrotracBELSopr-Aqua3 adsorption apparatus with a water circulator bath. Coverage-dependent heats of organic vapor adsorption data was collected with an adsorption-calorimetric joint instrument constituted by MicrotracBEL Belsorp-MAXII and KEP ChemStar (ADS-CAL). Anhydrous solvents were used for vapor adsorption, which degassed at least five times before isotherm collection.

To better demonstrate the host-guest relationship in PFC-76-NH_2 during adsorption, the measuring unit of gas uptake can be converted to mol/mol according to the following equation:

$$\boldsymbol{n_{ads}} = \frac{\boldsymbol{V_a m_{\mathrm{PFC-76-NH2}}}}{\boldsymbol{V_m}},$$

where n_{ads} represents the uptake after conversion, and $m_{\mathrm{PFC\text{-}76\text{-}NH2}}$ represents the molar mass of PFC-76-NH_2.

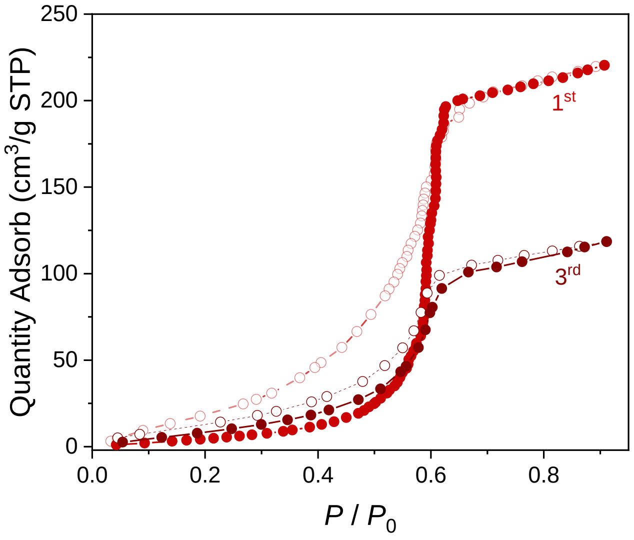

Figure S36. PFC-76 tested the change of adsorption capacity after one and three rounds of water adsorption.

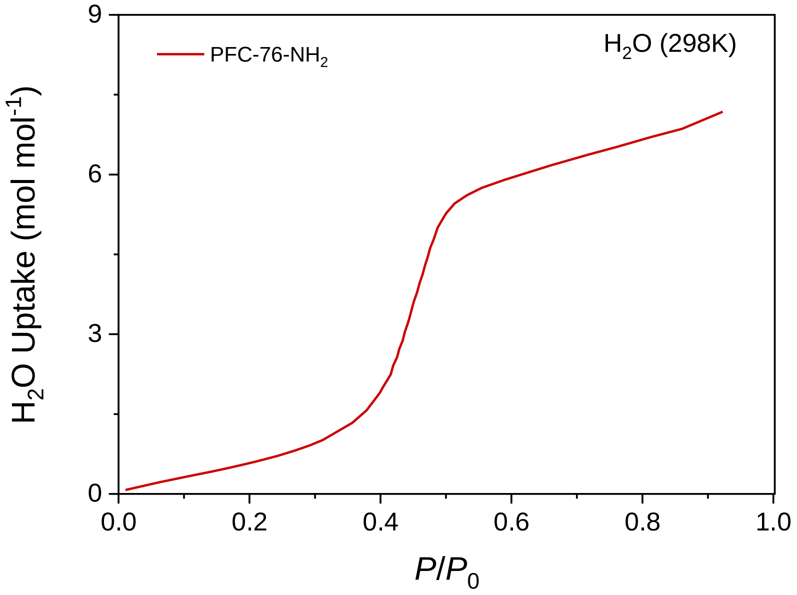

Figure S37. 298 K H_2O isotherms for PFC-76-NH_2.

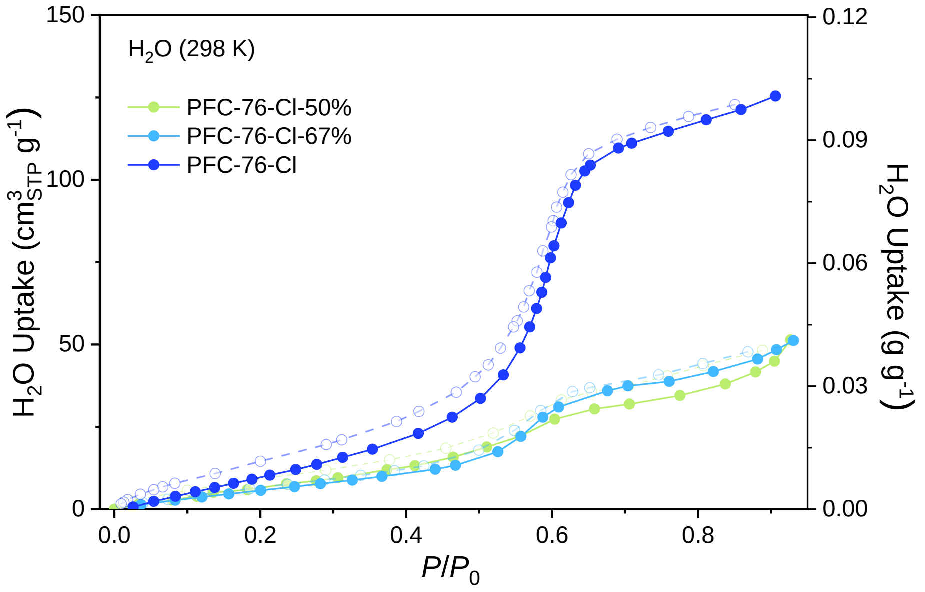

Figure S38. 298 K H_2O isotherms for a series of MTV PFC-76-Cl-*y* (*y* = 50%, 67%, and 100%) samples.

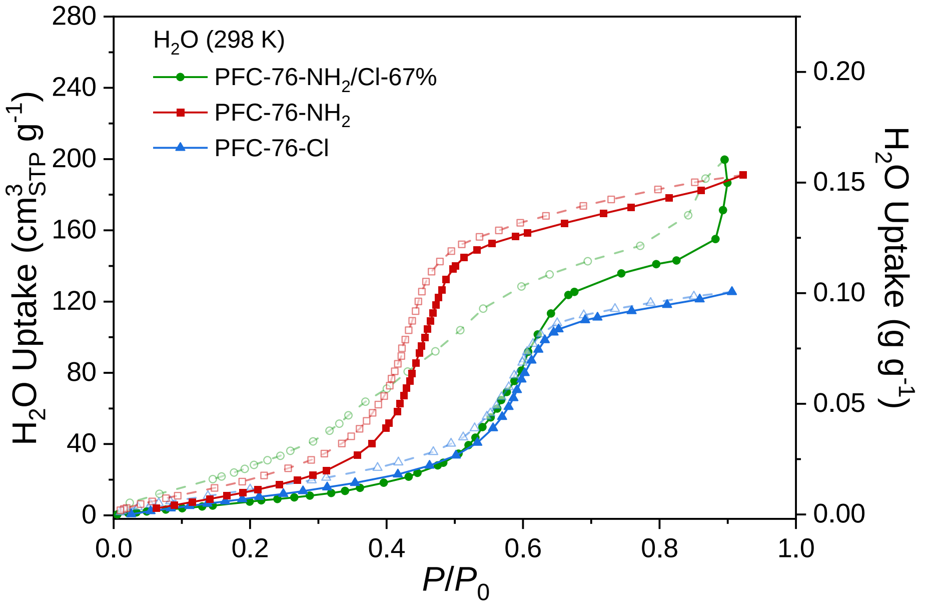

Figure S39. 298 K H_2O isotherms for MTV PFC-76-NH_2/Cl-67% sample.

Table S2. Water harvest properties for MTV functionality in HOFs

Structure	Water uptake ($g\ g^{-1}$)	0.5 water uptake ($g\ g^{-1}$)	Inflection point (P/P_0)	Work capacity ($g\ g^{-1}$)
PFC-76	0.178	0.089	0.6	0.013
PFC-76-NH_2-50%	0.158	0.079	0.48	0.086
PFC-76-NH_2-67%	0.195	0.098	0.47	0.131
PFC-76-NH_2-80%	0.188	0.094	0.46	0.121
PFC-76-NH_2	0.153	0.076	0.45	0.112
PFC-76-Cl-50%	0.041	0.020	0.59	0.010
PFC-76-Cl-67%	0.041	0.020	0.57	0.010
PFC-76-Cl	0.10	0.05	0.58	0.020
PFC-76-NH_2/Cl-67%	0.160	0.08	0.62	0.013

Work capacity: The V_{ads} on $P/P_0 = 0.5$ subtract V_{des} on $P/P_0 = 0.1$

Interaction point is the pressure where the water uptake is a half of the maximum water adsorption capacity.[4]

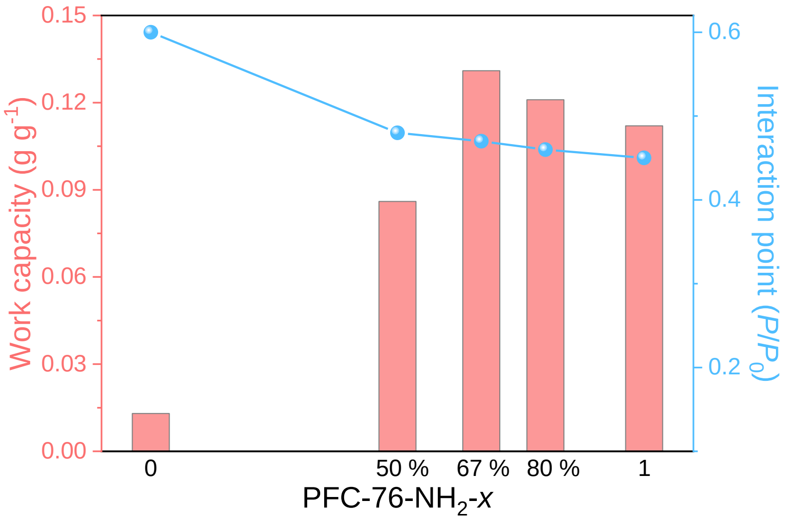

Figure S40. Work capacity and interaction point on MTV PFC-76-NH_2-*x* (*x* =0, 50%, 67%, 80%, and 100%).

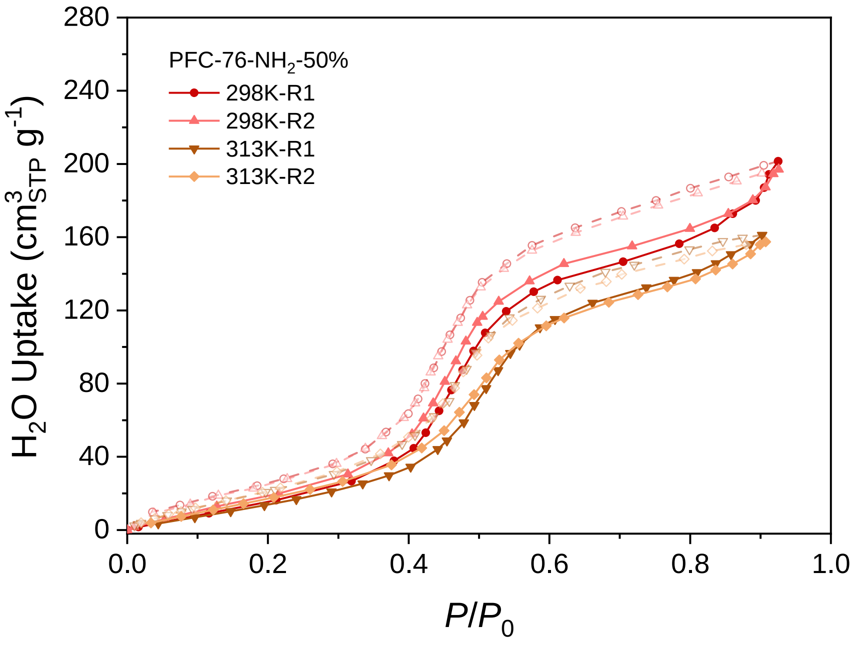

Figure S41. H_2O adsorption of PFC-76-NH_2-50% at 298 K and 313 K.

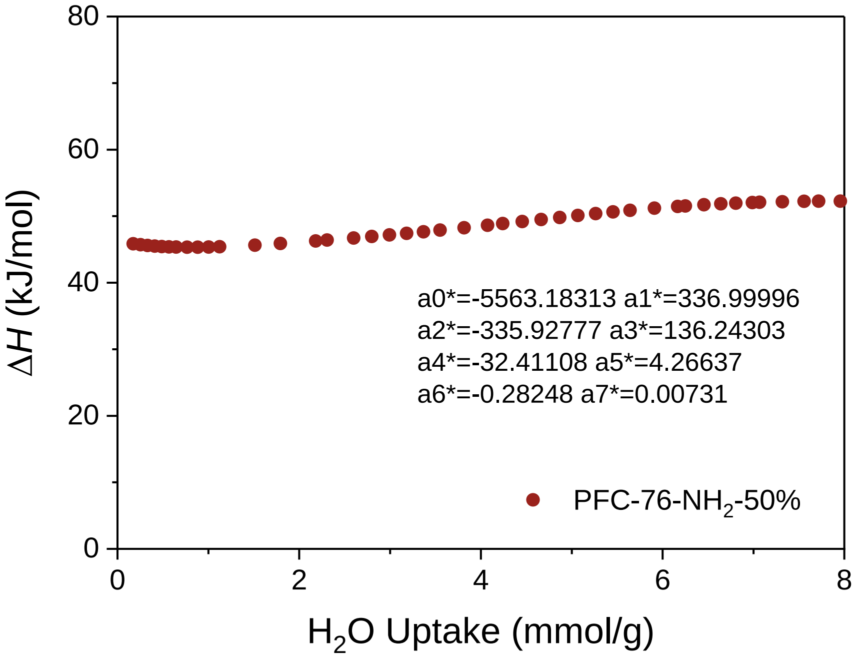

Figure S42. The adsorption heat of PFC-76-NH_2-50% was calculated using the Wiley method simulation.

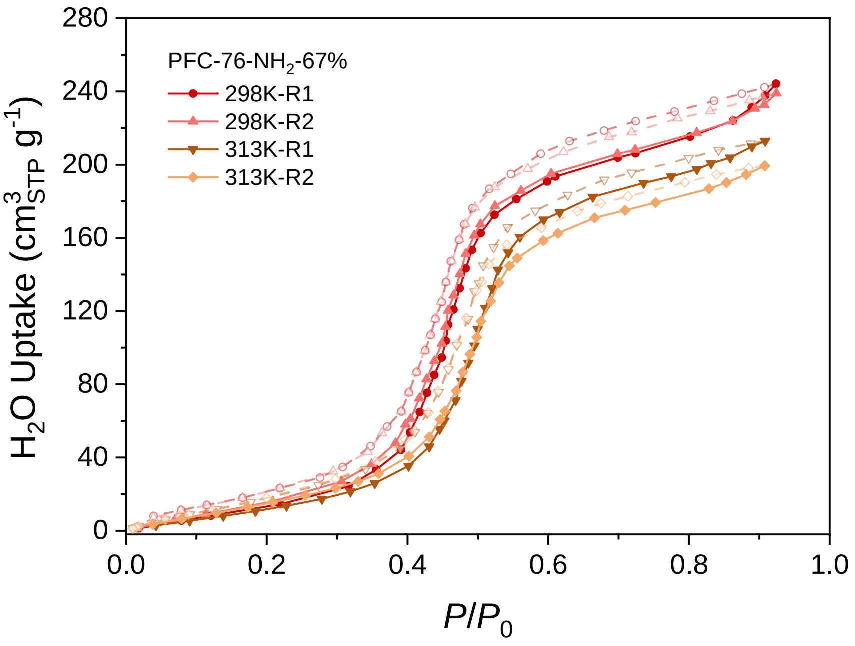

Figure S43. H_2O adsorption of PFC-76-NH_2-67% at 298K and 313 K.

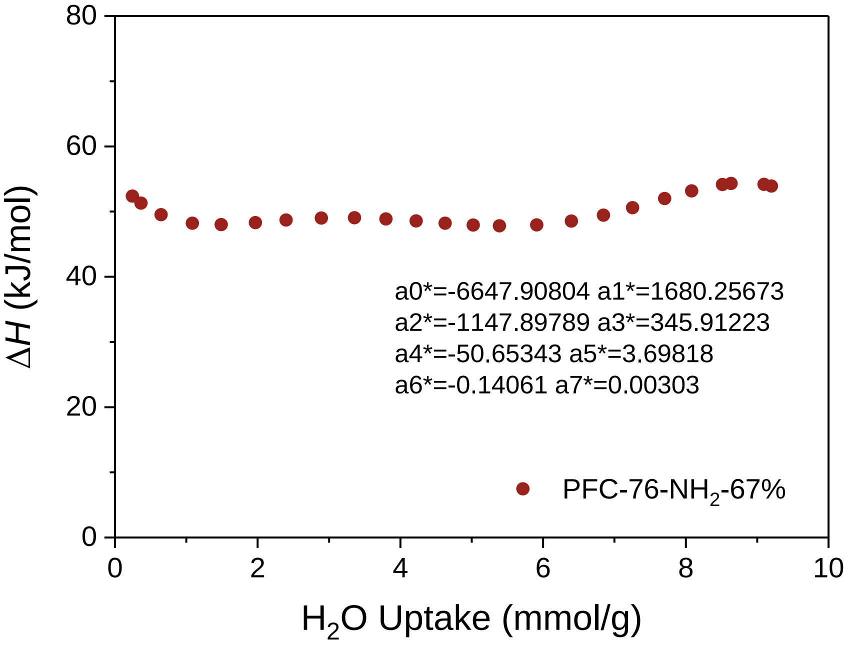

Figure S44. The adsorption heat of PFC-76-NH_2-67% was calculated using the Wiley method simulation.

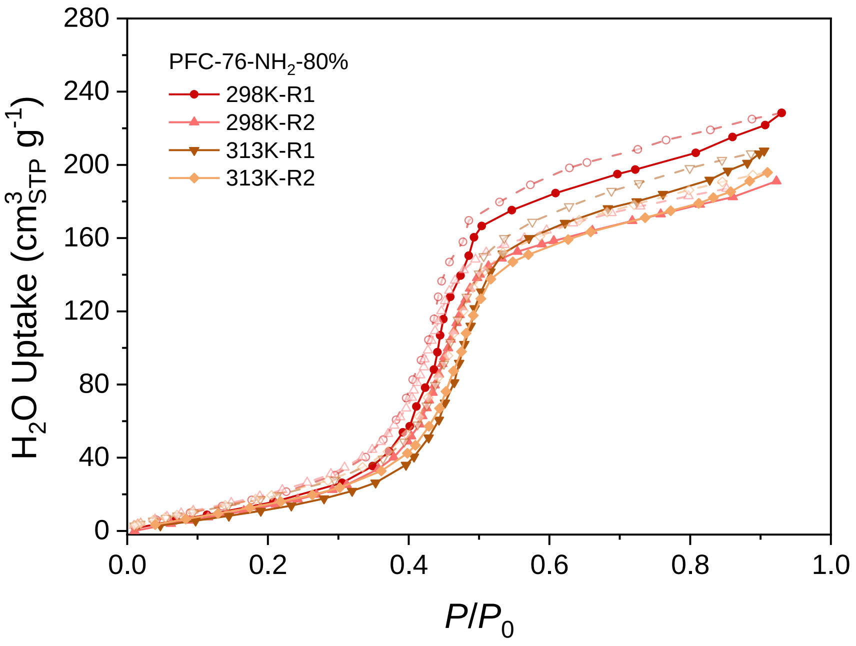

Figure S45. H_2O adsorption of PFC-76-NH_2-80% at 298 K and 313 K.

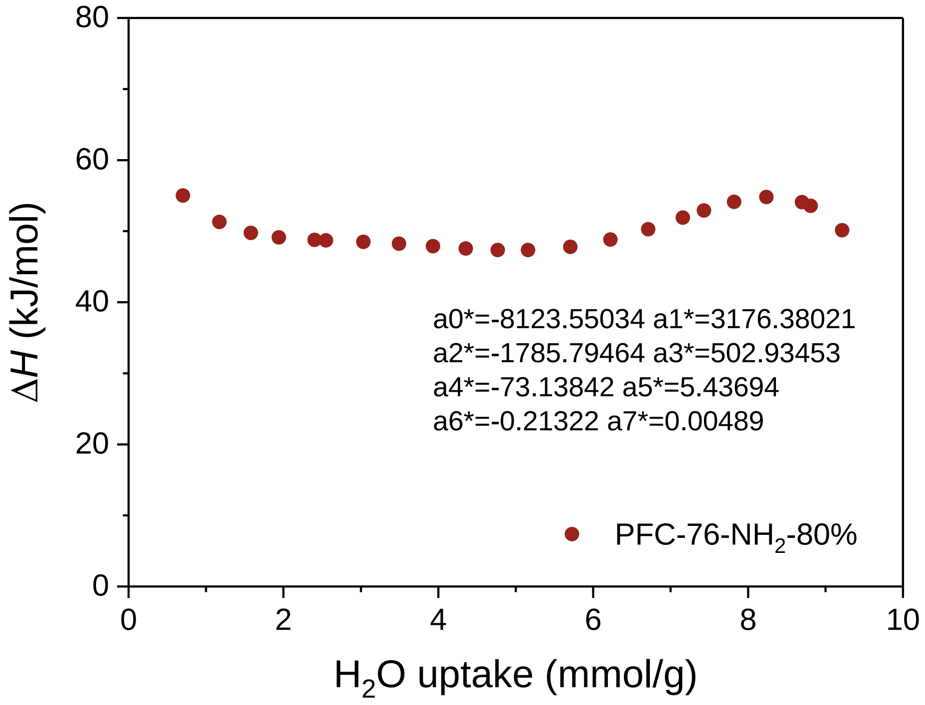

Figure S46. The adsorption heat of PFC-76-NH_2-80% was calculated using the Wiley method simulation.

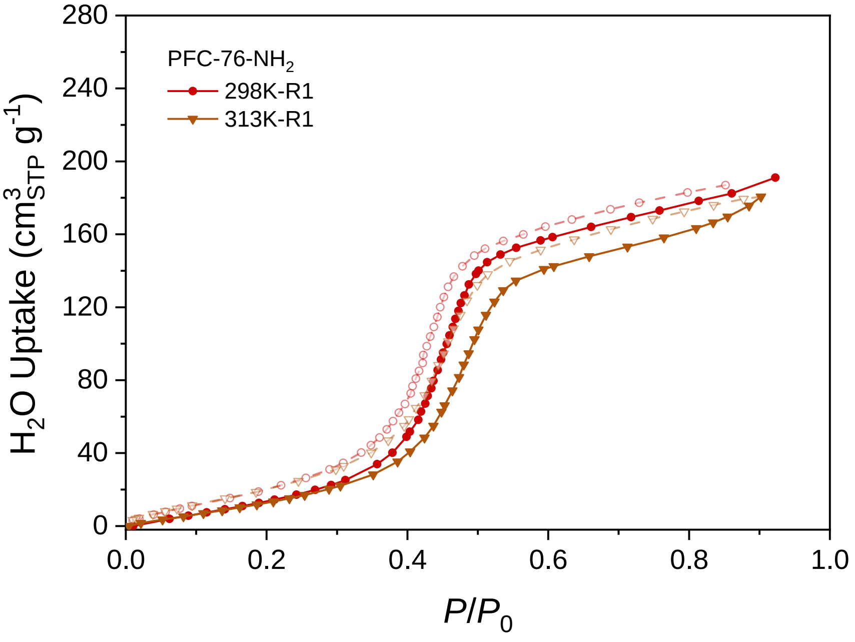

Figure S47. H_2O adsorption of PFC-76-NH_2 at 298 K and 313 K.

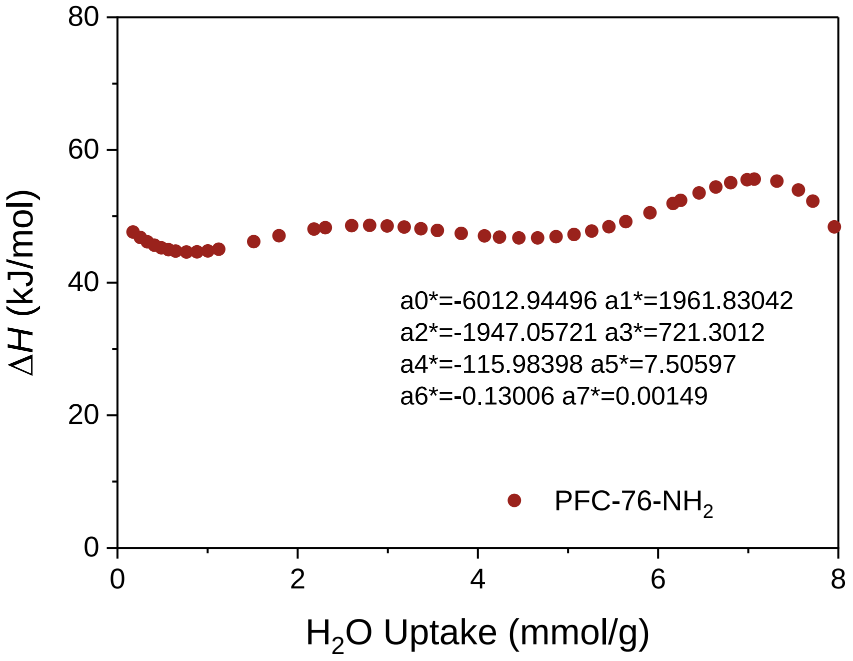

Figure S48. The adsorption heat of PFC-76-NH_2 was calculated using the Wiley method simulation.

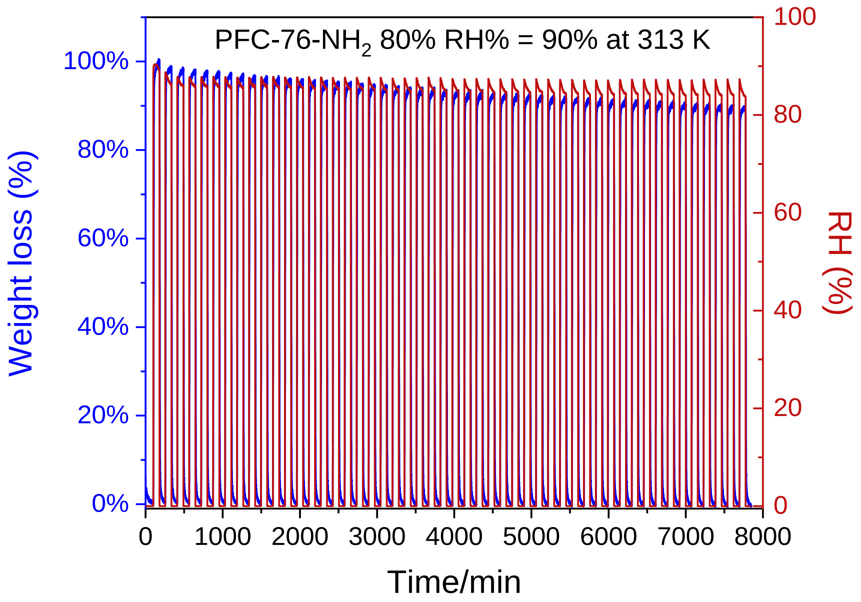

Figure S49. Recyclability (50 cycles) of PFC-76-NH_2-80% at humidity levels ranging from 0 to 90% at 313 K. After 50 cycles, PFC-76-NH_2-80% retained 89.5% of its water capture capacity. PFC-76-NH_2-67% is more stable than PFC-76-NH_2-80%, probably due to fewer H_2O adsorption sites in structure and reduced mechanical deformation during the adsorption-desorption processes.

Section S6. Theorical Calculation and Molecular Simulation

Panoramic View of Conformational Energy: The conformational search are Molclus software[5] was used to calculate the different conformational phase single-point energy using MMFF44 force filed[6] embedded Open Babel software.[7]

Independent gradient model based on Hirshfeld partition of molecular density (IGMH) analyses. The independent gradient model based on Hirshfeld partition of molecular density (IGMH) method[8] reported by Lu et al. in 2022 was used to explore the framework-framework interactions. IGMH method is an improved version of independent gradient mode (IGM)[9] which replaces the free-state atomic densities involved in the IGM method with the atomic densities derived by Hirshfeld partition of actual molecular electron density. The $\boldsymbol{\delta g}$ function are defined as the difference between IGM type of density gradient ($\boldsymbol{g^{IGM}}$) and the gradient of promolecular density ($\boldsymbol{g}$). This parameter has a positive correlation with the strength of intermolecular interactions within an interatomic interaction region and can thus be used to measure the relative strength of these interactions[10].

The intermolecular interactions can be expressed as[8]

$$\boldsymbol{\delta g^{inter}(r) = g^{IGM,inter}(r) - g^{inter}(r)}$$

with

$$\boldsymbol{g^{inter}(r) = \left|\sum_{A}\sum_{i\epsilon A} \nabla\rho_i^{free}(r)\right|}$$

where $\boldsymbol{r}$ represents the Cartesian coordinate vector, $\boldsymbol{A}$ loops over all fragments, $\boldsymbol{i}$ loops all atoms in the corresponding fragment and $\boldsymbol{\rho_i^{free}}$ represents the spherically averaged density of atom $\boldsymbol{i}$ in its free state. The IGMH analyses are CP2K software[11] was used to conduct the DFT calculations to get the wavefunction files using PBE[12]-D3(BJ)[13] exchange-correlation density functional with the DZVP-MOLOPT-SR-GTH basis set.[14]

Intercepting the adjacent three-packing structure of the four PFC-76-X (X = H, NH_2, Cl) links for IGM calculations, we focus only on the intermolecular interactions, denoted as δg-inter. and decompose the inter-framework interactions into the contribution of each atom, denoted as δg-index, the larger the δg-index, the larger the contribution of the atom to the interactions. And VMD is used to color the atoms by δg-index to show the structure.

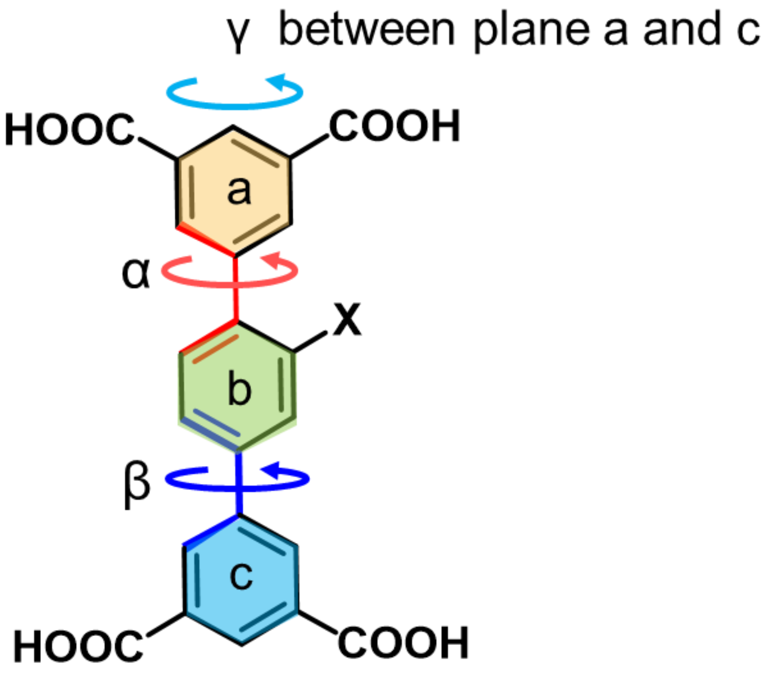

Figure S50. Dihedral angles between the triphenylene core.

Table S3. Dihedral angles between the triphenylene core

Structure	α / °	β / °	γ / °
PFC-76	31.59	36.02	4.83
PFC-76-NH_2-as	42.51	-38.31	5.38
PFC-76-NH_2	43.51	-15.81	30.51
PFC-76-NH_2-H_2O	40.30	-42.31	1.17
PFC-76-Cl	41.26	-41.26	0

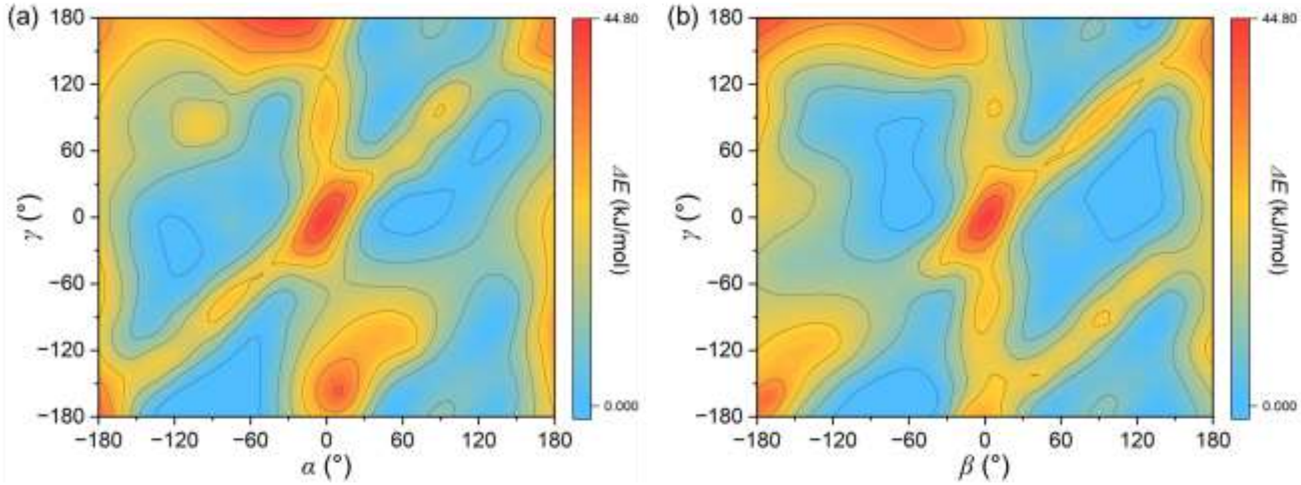

Figure S51.The rotational energy landscape of TPTCA with the dihedral angles α and γ (a), β and γ (b).

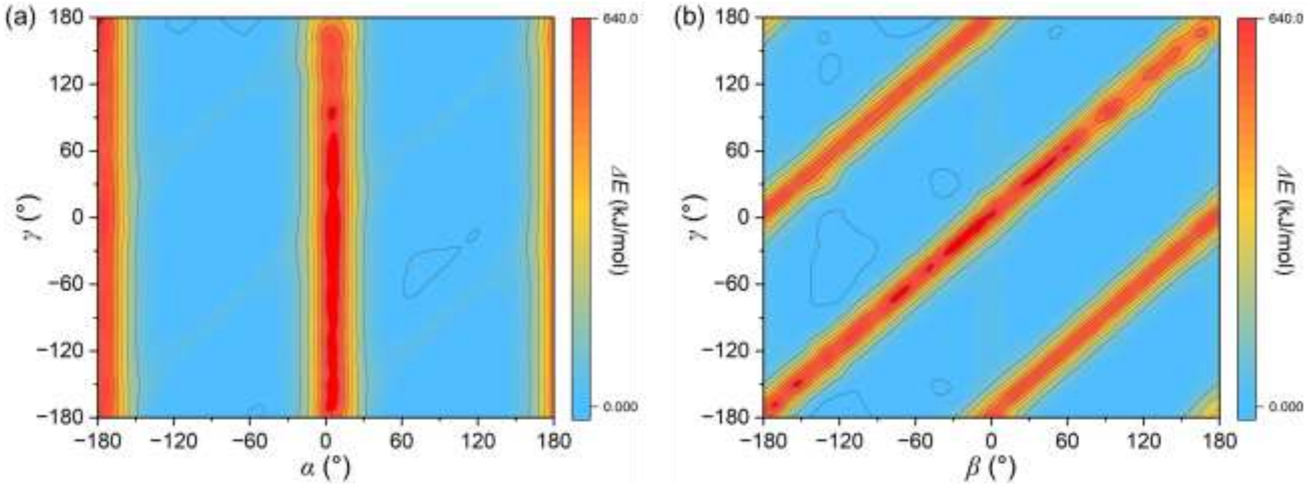

Figure S52.The rotational energy landscape of TPTCA-NH_2 with the dihedral angles *α* and *γ* (a), *β* and *γ* (b).

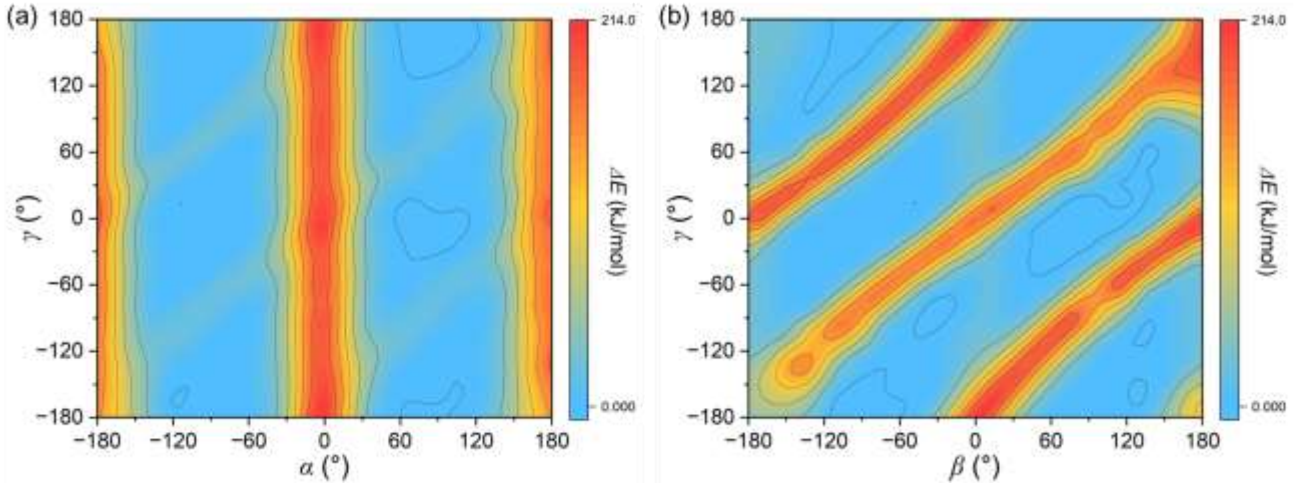

Figure S53.The rotational energy landscape of TPTCA-Cl with the dihedral angles *α* and *γ* (a), *β* and *γ* (b).

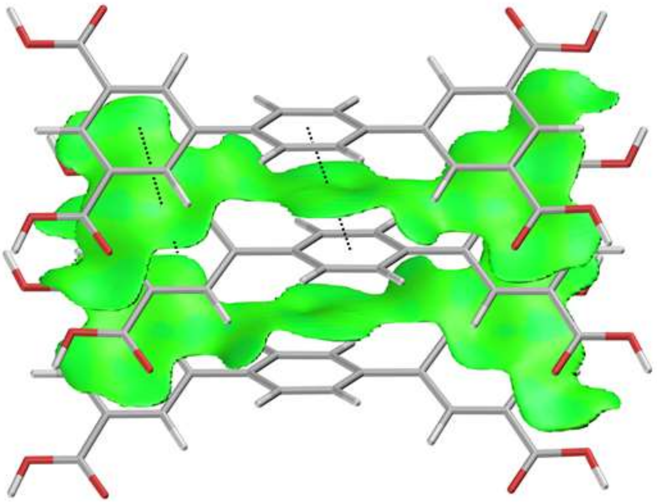

Figure S54. *Sign(δ₂)ρ* colored isosurfaces of δg^{inter} = 0.005 a.u. of host-host interactions via IGMH method for PFC-76.

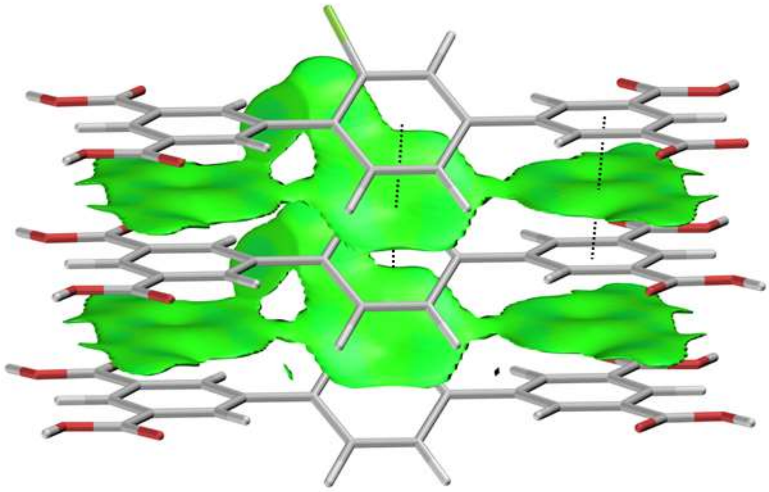

Figure S55. *Sign(λ_2)ρ* colored isosurfaces of δg^{inter} = 0.005 a.u. of host-host interactions via IGMH method for PFC-76-Cl.

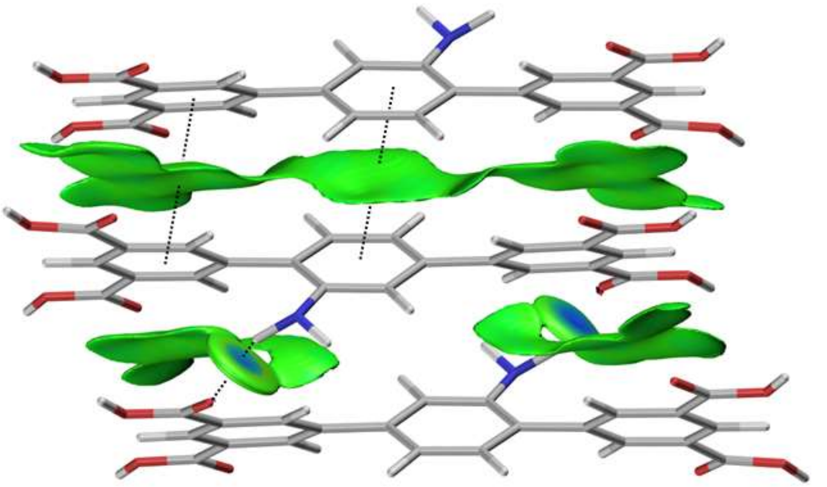

Figure S56. *Sign*$(\lambda_2)\rho$ colored isosurfaces of δg^{inter} = 0.005 a.u. of host-host interactions via IGMH method for PFC-76-NH_2-as.

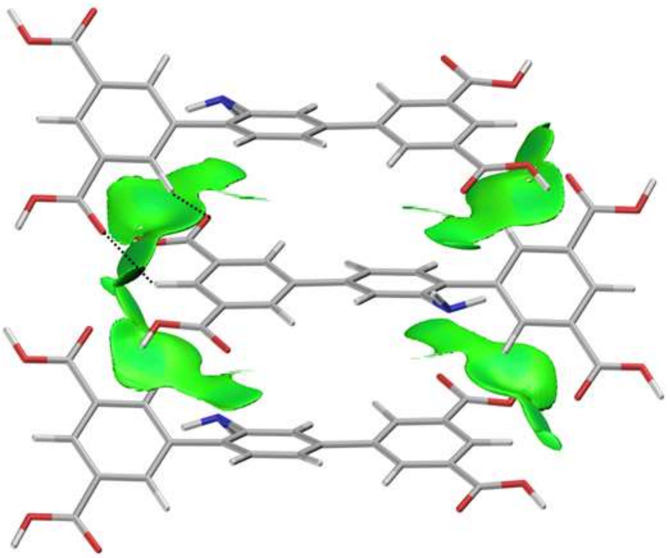

Figure S57. $Sign(\lambda_2)\rho$ colored isosurfaces of δg^{inter} = 0.005 a.u. of host-host interactions via IGMH method for PFC-76-NH_2.

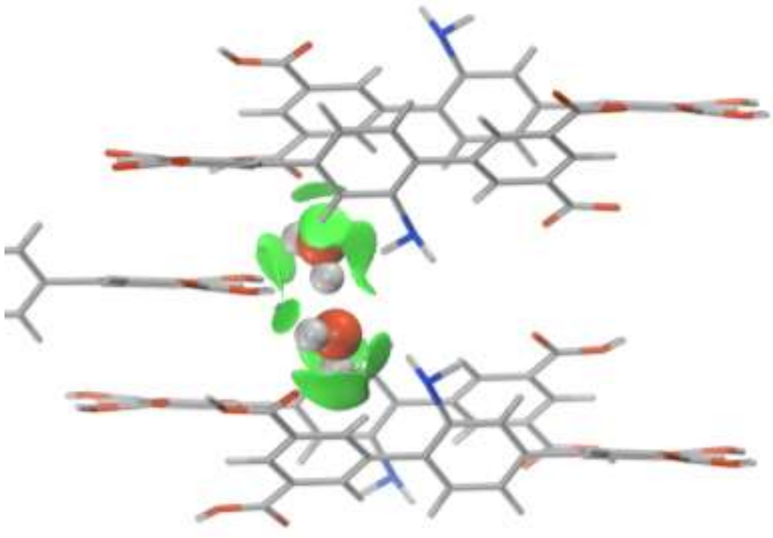

Figure S58. *Sign(λ₂)ρ* colored isosurfaces of δg^{inter} = 0.005 a.u. of host-guest interactions via IGMH method for PFC-76-NH_2.

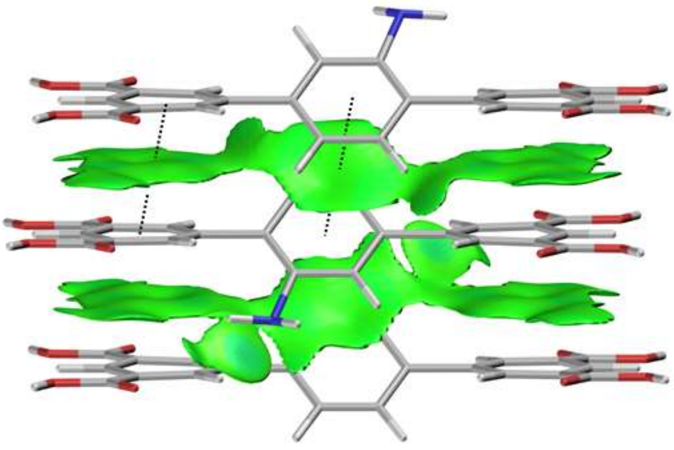

Figure S59. *Sign(λ₂)ρ* colored isosurfaces of δg^{inter} = 0.005 a.u. of host-host interactions via IGMH method for PFC-76-NH_2-H_2O

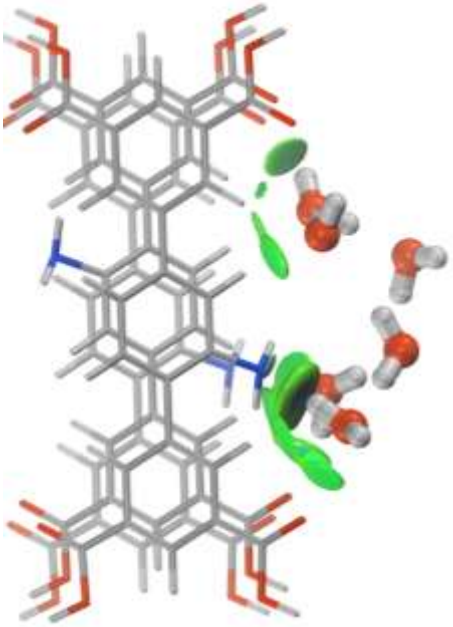

Figure S60. *Sign(λ₂)ρ* colored isosurfaces of δg^{inter} = 0.005 a.u. of host-guest interactions via IGMH method for PFC-76-NH_2-H_2O

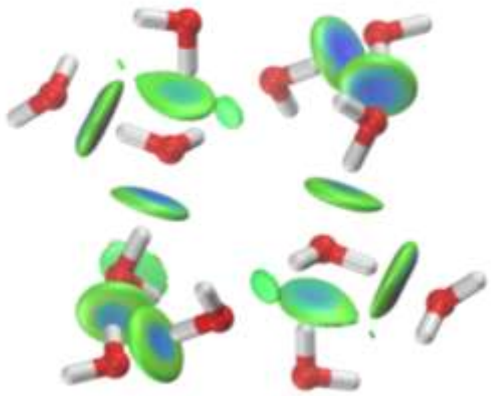

Figure S61. *Sign(λ₂)ρ* colored isosurfaces of δg^{inter} = 0.005 a.u. of guest-guest interactions via IGMH method for PFC-76-NH_2-H_2O

References

S1. Park, J., Wang, Z. U., Sun, L.-B., Chen, Y.-P. & Zhou, H.-C. Introduction of Functionalized Mesopores to Metal–Organic Frameworks via Metal–Ligand–Fragment Coassembly. *J. Am. Chem. Soc.* **2012**, *134*, 20110–20116

S2. Sheldrick, G.M. A short history of SHELX. *Acta Cryst*. **2008**, *A64*, 112.

S3. Qin, W. K.; Si, D. H.; Yin, Q.; Gao, X. Y.; Huang, Q. Q.; Feng, Y. N.; Xie, L.; Zhang, S.; Huang, X. S.; Liu, T. F.; et al. Reticular Synthesis of Hydrogen‐Bonded Organic Frameworks and Their Derivatives via Mechanochemistry. *Angew. Chem. Int. Ed.* **2022**, *61,* e202312393.

S4. Brahma, D.; Dwarkanath, N.; Balasubramanian, S. Insights into Isotherm Step and Pore-Filling Mechanisms for Water Adsorption in Covalent Organic Frameworks. *Chem. Mater.* **2024**, *36,* 5651–5660

S5. Lu, T. Molclus Program, Version 1.12, http://www.keinsci.com/research/molclus.html (accessed Nov 6, 2024)

S6. (a) Halgren, T. A. Merck molecular force field. I. Basis, form, scope, parameterization, and performance of MMFF94. *J. Comput. Chem*., **1996**, *17*, 490-519. (b) Halgren, T. A. Merck molecular force field. II. MMFF94 van der Waals and electrostatic parameters for intermolecular interactions. *J. Comput. Chem*., **1996**, *17*, 520-552. (c) Halgren, T. A. A. Merck molecular force field. III. Molecular geometries and vibrational frequencies for MMFF94. *J. Comput. Chem*., **1996**, *17*, 553-586. (d) Halgren, T. A.; Nachbar, R.B. Merck molecular force field. IV. conformational energies and geometries for MMFF94. *J. Comput. Chem*., **1996**, *17*, 587-615. (e) Halgren, T. A. Merck molecular force field. V. Extension of MMFF94 using experimental data, additional computational data, and empirical rules. *J. Comput. Chem*., **1996**, *17*, 616-641.

S7. O'Boyle, N.M.; Banck, M.; James, C.A. Morley, C.; Vandermeersch T.; Hutchison, R.H. Open Babel: An open chemical toolbox. *J. Cheminform.,* **2011**, *3*, 33.

S8. Lu, T.; Chen, Q. Independent gradient model based on Hirshfeld partition: A new method for visual study of interactions in chemical systems. *J. Am. Chem. Soc.* **2022**, *43*, 539-555.

S9. Lefebvre, C.; Khartabil, H.; Boisson, J.-C.; Contreras-García, J.; Piquemal, J.-P.; Hénon, E. The Independent Gradient Model: A New Approach for Probing Strong and Weak Interactions in Molecules from Wave Function Calculations. *Chem. Phys. Chem.* **2018**, *19*, 724-735.

S10. Lefebvre, C.; Rubez, G.; Khartabil, H.; Boisson, J.-C.; Contreras-García, J.; Hénon, E. Accurately extracting the signature of intermolecular interactions present in the NCI plot of the reduced density gradient versus electron density. *Phys. Chem. Chem. Phys.* **2017**, *19*, 17928-17936.

S11. Kühne, T. D.; Iannuzzi, M.; Del Ben, M.; Rybkin, V. V.; Seewald, P.; Stein, F.; Laino, T.; Khaliullin, R. Z.; Schütt, O.; Schiffmann, F.; et al. CP2K: An electronic structure and molecular dynamics software package - Quickstep: Efficient and accurate electronic structure calculations. *J.Chem. Phys.* **2020**, *152*, 194103.

S12. Perdew, J. P.; Burke, K.; Ernzerhof, M. Generalized Gradient Approximation Made Simple. *Phys Rev Lett* **1996**, *77* (18), 3865-3868.

S13. Grimme, S.; Ehrlich, S.; Goerigk, L. Effect of the damping function in dispersion corrected density functional theory. *J. Comput. Chem.* **2011**, *32*, 1456-1465.

S14. VandeVondele, J.; Hutter, J. Gaussian basis sets for accurate calculations on molecular systems in gas and condensed phases. *J.Chem. Phys.* **2007**, *127*, 114105.
